# Lactylation Reprogramming in the Bone Infection Microenvironment Identifies PGK1 K361 as a Potential Therapeutic Target for Osteogenic Dysfunction

**DOI:** 10.1002/advs.202523583

**Published:** 2026-01-28

**Authors:** Han‐jun Qin, Si‐ying He, Ting‐hui Xiao, Da‐mao Dai, Xin‐jia Hu, Nan Jiang

**Affiliations:** ^1^ Division of Orthopaedic Trauma Department of Orthopaedic Surgery Shenzhen People's Hospital (The First Affiliated Hospital, Southern University of Science and Technology; The Second Clinical Medical College, Jinan University) Shenzhen China; ^2^ Shenzhen Key Laboratory of Musculoskeletal Tissue Reconstruction and Function Restoration Department of Orthopaedic Surgery Shenzhen People's Hospital (The First Affiliated Hospital, Southern University of Science and Technology; The Second Clinical Medical College, Jinan University) Shenzhen China; ^3^ Department of Plastic and Cosmetic Surgery Shenzhen People's Hospital (The First Affiliated Hospital, Southern University of Science and Technology; The Second Clinical Medical College, Jinan University) Shenzhen China; ^4^ Department of Trauma Emergency Center Ganzhou Hospital‐Nanfang Hospital Southern Medical University Ganzhou China; ^5^ Division of Orthopaedics and Traumatology Department of Orthopaedics Nanfang Hospital Southern Medical University Guangzhou China

**Keywords:** bone infection, lysine lactylation, phosphoglycerate kinase 1 (PGK1), mitophagy, ferroptosis

## Abstract

Bone infections pose a significant global challenge in orthopedics, often leading to poor bone healing, limb dysfunction, and the need for additional surgeries. Lysine lactylation (Kla) has emerged as a novel post‐translational modification, garnering considerable research attention. However, its role in bone infection remains unclear. In this study, results show that Kla levels are significantly higher in the bone tissues of infected patients than in the uninfected controls. Global Kla quantitative proteomics identified 491 Kla sites on 201 proteins, each with distinct expression patterns in bone tissue. Phosphoglycerate kinase 1 (PGK1), a key glycolytic enzyme, undergoes lactylation at residue K361. By designing adenoviral vectors that mimic either the lactylated or delactylated forms of this site and employing adeno‐associated viruses to specifically target osteoblasts, in vitro and in vivo studies suggest that modifying PGK1 at K361 through Kla may offer a promising strategy for treating infection‐induced osteogenic dysfunction. Integrating patient proteomic data further reveals and validates a novel mechanism: PGK1 K361 lactylation activates VDAC3, triggering FtMt/PINK1/Parkin‐mediated mitophagy and inducing ferroptosis in osteoblasts. Collectively, these findings provide new mechanistic insights into osteogenic impairment during bone infection and suggest that PGK1 K361 lactylation is a promising intervention target.

## Graphical abstract

*Staphylococcus aureus (S. aureus)* infection creates a high‐lactate microenvironment, promoting p300‐mediated lactylation of PGK1 at lysine 361 (K361). Lactylated PGK1 translocates to the mitochondrial outer membrane and interacts with VDAC3. This interaction triggers FtMt downregulation, iron accumulation, and excessive PINK1/Parkin‐mediated mitophagy, ultimately driving lipid peroxidation and ferroptosis in osteoblasts, leading to bone loss.

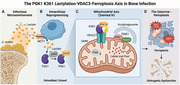

## 1 Introduction

Bone infection (osteomyelitis) continues to present a substantial and costly challenge within orthopedics. This condition is defined by sustained microbial colonization, biofilm formation, chronic inflammation, and a hostile microenvironment that collectively impede bone regeneration [1]. Despite advancements in care—including meticulous debridement, staged reconstruction, administration of local and systemic antibiotics, and application of bioactive materials—clinical outcomes are often limited by infection‐induced osteogenic dysfunction, delayed union or nonunion, and an elevated risk of reoperation [2]. These factors contribute to extended hospitalization, reduced patient functionality, and increased socioeconomic burden [3]. While surgical innovation and antimicrobial stewardship continue to progress, a central unmet need remains: the development of molecularly targeted strategies that directly protect and restore the osteogenic potential of infected bones.

Converging evidence suggests that cellular metabolism functions not only as a source of energy but also as a key regulator of lineage commitment and stress adaptation in skeletal cells [4]. During infection and repair, osteoblasts, osteoclasts, and infiltrating immune cells undergo profound metabolic reprogramming; these immunometabolic shifts within the bone microenvironment, such as those observed in macrophage immunometabolism, influence inflammatory responses and reparative mechanisms that ultimately affect osteoblast function [5]. Factors including hypoxia, inflammatory cytokines, bacterial metabolites, and nutrient competition collectively enhance glycolytic flux and lactate accumulation within the infected bone niche [6]. Lactate, previously viewed solely as a metabolic product, now emerges as both a carbon source and a signaling molecule, influencing immune modulation, angiogenesis, and matrix remodeling [7]. However, the precise mechanisms by which these infection‐associated metabolic shifts translate into durable changes in osteoblast function and survival remain poorly defined.

One promising approach for further investigation involves the recently defined post‐translational modification (PTM), lysine lactylation (Kla) [8]. In 2019, lactylation was established as a direct biochemical link between glycolytic metabolism and protein regulation [8]. Histone lactylation has been shown to connect intracellular lactate levels to chromatin dynamics and gene expression patterns [9]. Subsequent research has expanded this landscape to include non‐histone substrates, implicating lactylation in immunity, inflammation, tumor biology, and tissue repair [10]. Nevertheless, within the skeletal field—particularly in bone infection—lactylation in osteoblast biology remains an emerging area of exploration. Compared to other acyl‐lysine PTMs, such as acetylation and succinylation [11, 12], the enzymology, substrate specificity, and functional implications of lactylation in osteogenic cells remain underexplored, leaving a notable gap in mechanistic understanding at the intersection of infection‐driven metabolism and bone regeneration.

Glycolytic enzymes are promising targets for lactylation‐mediated regulation as they sit at key intersections of energy production, biosynthesis, and cellular signaling. Phosphoglycerate kinase 1 (PGK1) catalyzes the first ATP‐generating step in glycolysis and plays several non‐canonical roles beyond metabolism, including modulating stress responses and facilitating interorganelle communication [13]. PTMs of these enzymes can rapidly alter metabolic flux, enzyme activity, subcellular localization, and protein–protein interactions, thus influencing cellular outcomes under stress [14]. In osteoblasts challenged by infection, inflammation, and hypoxia, PGK1 may act as a central molecular hub linking metabolic signals with cell survival or death [15]. However, the specific PTMs of PGK1 in osteoblasts—particularly lactylation at defined lysine residues and their effect on osteogenic homeostasis during infection—remain largely undefined.

In this study, we identified a lysine lactylation at PGK1 K361 and showed that it reshaped the stress response in osteoblasts. Specifically, lactylation at K361 remodels the PGK1 protein‐protein interaction network and regulates VDAC3. This mechanism may provide a coherent explanation for the clinical observation that infection compromises osteogenesis even after bacterial loads are reduced: a PTM encoded, cell‐intrinsic program could lock osteoblasts into a vulnerable state. Understanding the associated pathway could provide a metabolic epigenetic framework for bone infection and reveal actionable molecular targets for precise prevention and therapy.

## 2 Results

### 2.1 Elevated Lactate in the Infectious Bone Microenvironment Induced Protein Lactylation and Impaired Osteoblast Function

**FIGURE 1:**
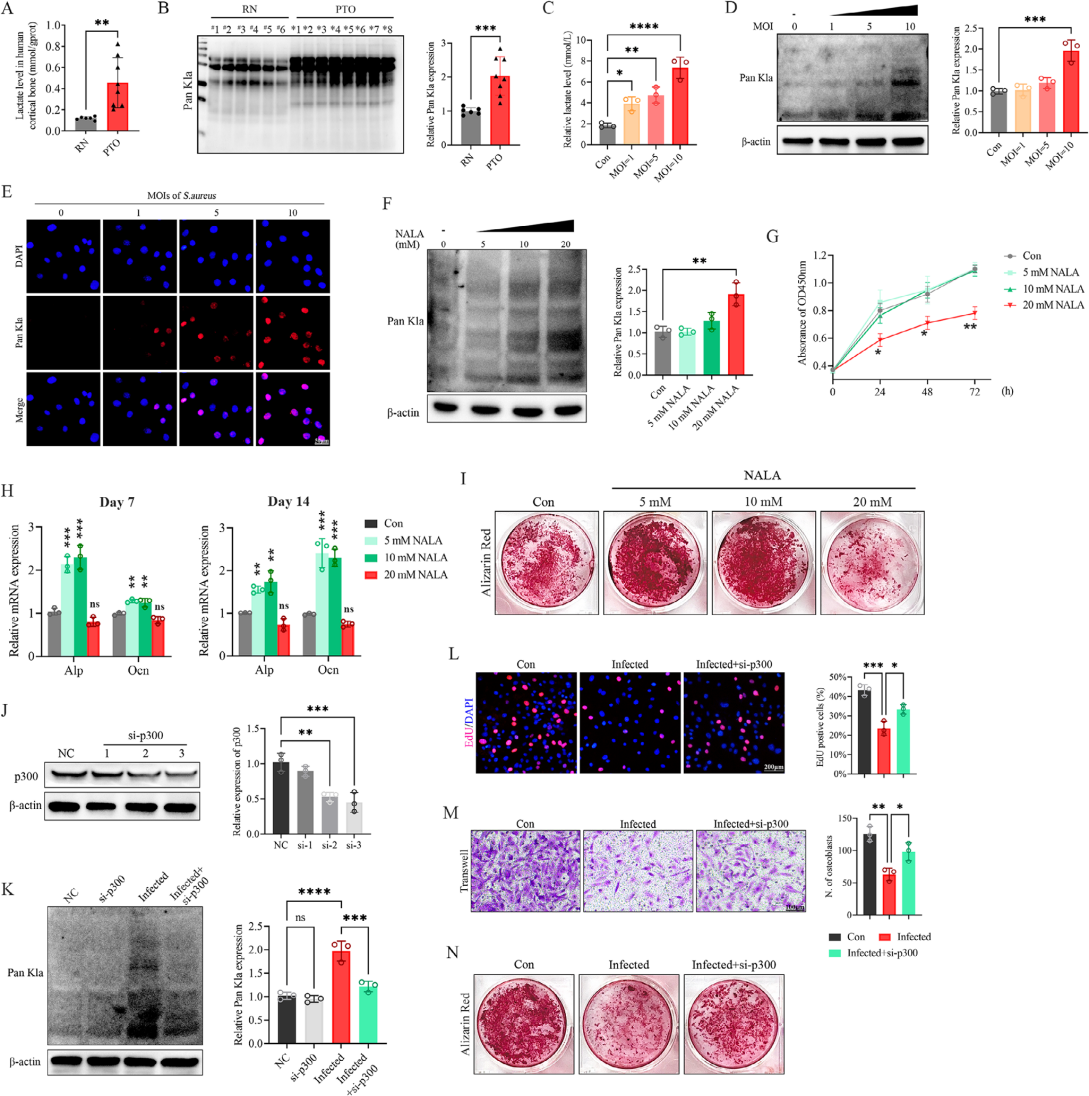
Elevated lactate levels in the infectious bone microenvironment increased protein lactylation and impaired osteogenic function. (A) Lactate levels in human bone tissues (RN, *n* = 6; PTO, *n* = 8). (B) Western blot and quantification of global protein lactylation (Kla) in human bone tissues (RN, *n* = 6; PTO, *n* = 8). (C) Extracellular lactate levels in murine preosteoblast cell line MC3T3‐E1 infected with *S. aureus* at varying MOIs. (D) Western blotting and quantification of global Kla in osteoblasts under increasing MOIs. (E) Representative images of Kla immunocytochemistry (ICC) in osteoblasts under MOIs; Scale bar, 50 µm. (F) Western blotting and quantification of global Kla in osteoblasts under increasing sodium l‐lactate (NALA) concentrations. (G) Effect of NALA on osteoblast proliferation. (H) RT‐qPCR analysis of alkaline phosphatase (*Alp;* Day 7) and osteocalcin (*Ocn;* Day 14) mRNA levels during osteogenic induction with NALA. (I) Representative images of Alizarin Red S staining at day 28 under NALA. (J) Western blotting and quantification of p300 knockdown efficiency. (K) Western blotting and quantification of global Kla after p300 knockdown. (L) Representative images and quantification of the EdU assay; Scale bar, 200 µm. (M) Representative images and quantification of the Transwell assay; Scale bar, 100 µm. (N) Representative images of Alizarin Red S staining on day 28. RN, relatively normal; PTO, post‐traumatic osteomyelitis; MOI, multiplicity of infection. Data represent the mean ± SEM, *n* = 3 per group (C–N). Statistical significance was assessed using the unpaired Student's *t*‐test (A,B), one‐way ANOVA with Dunnett's multiple comparisons test (C,D,F,H, J–M), and two‐way ANOVA with Dunnett's multiple comparisons test (G). All groups were compared with the control or NC groups; the groups without significant markers were considered not significant. ^*^ *p* < 0.05, ^**^ *p* < 0.01, ^***^ *p* < 0.001, ^****^ *p* < 0.0001, ns: no significance.

In clinical bone tissue, the post‐traumatic osteomyelitis (PTO) group exhibited higher lactate levels (Figure 1A) and global protein Kla (Figure 1B), indicating these changes occur simultaneously during infection.

In vitro, murine preosteoblast cell line MC3T3‐E1 (osteoblast) cultures exposed to higher *Staphylococcus aureus* (*S. aureus*) multiplicities of infection (MOIs) had increased extracellular lactate (Figure 1C), with minimal pH change (ΔpH < 0.2; Figure S1B), suggesting that acidosis was not the primary driver. Infection also increased intracellular Kla levels (Figure 1D,E).

To determine whether lactate alone can elevate Kla, osteoblasts were exposed to increasing concentrations of sodium l‐lactate (NALA) under near‐constant pH (ΔpH < 0.2; Figure S1C), resulting in a dose‐dependent increase in Kla (Figure 1F). Functionally, 20 mm NALA reduced cell proliferation (Figure 1G), decreased the early osteogenic marker alkaline phosphatase (*Alp*) on day 7, and the late marker osteocalcin (*Ocn*) on day 14 during induction. Additionally, mineralized nodule formation was diminished on day 28 (Figure 1H,I). These findings align with previous observations that lower lactate concentrations favor osteogenic differentiation, whereas higher levels inhibit it.

The necessity of p300‐dependent lactylation in functional impairment was assessed by knocking down p300 in osteoblasts (Figure 1J), followed by infection at an MOI of 10. This approach reduced global Kla levels (Figure 1K) and significantly restored cell proliferation, migration, and osteogenic differentiation that had been inhibited by infection (Figure 1L–N). Taken together, these data suggest that during infection, excessive lactate reprograms osteoblasts via p300‐mediated protein lactylation, ultimately inhibiting their proliferation and differentiation.

### 2.2 Lactylome Profiling of Clinical Samples Identified a Metabolic Control Node

**FIGURE 2:**
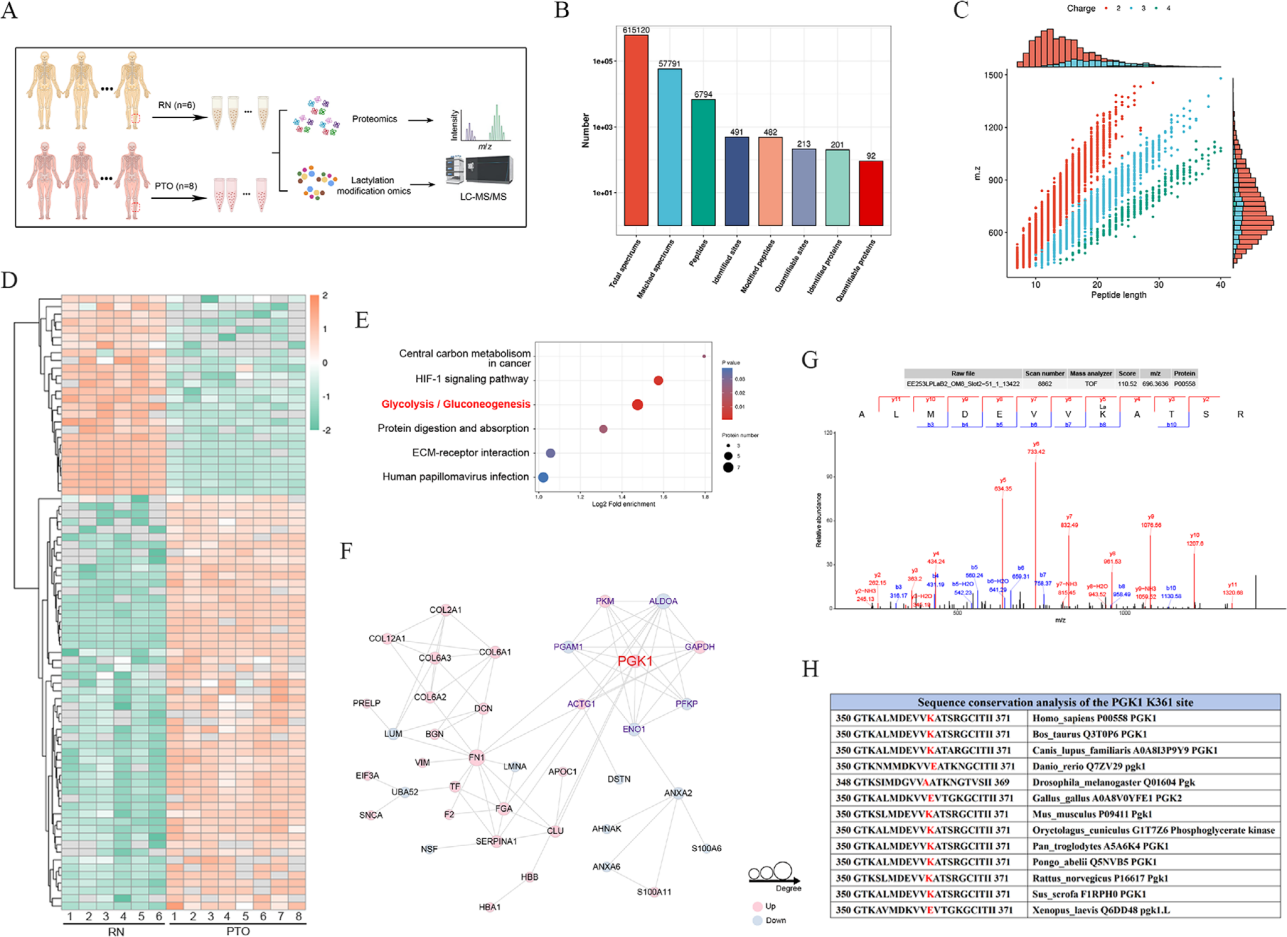
Lactylome profiling of clinical samples identifies a metabolic control node. (A) Workflow of integrated lysine lactylation proteomics and global proteomics on human bone tissues. (B) Summary statistics of identified/quantified Kla sites and proteins. (C) Peptide length distribution. (D) Heatmap of significantly altered Kla sites. (E) KEGG pathway enrichment bubble plot. (F) Protein–protein interaction (PPI) network of lactylated proteins based on the STRING database. (G) Representative MS/MS spectrum of a lactylated PGK1 peptide. (H) Cross‐species conservation of PGK1 K361.

Integrated lysine lactylation analysis and global proteomics were conducted on bone tissue samples from patients with PTO and healthy controls (Figure 2A). A total of 491 Kla sites were detected across 201 proteins, with quantitative data available for 213 sites spanning 92 proteins (Figure 2B). Quality control assessments confirmed that peptide lengths predominantly ranged from 7 to 20 amino acids, aligning with expectations for tryptic digestion and standard mass spectrometry (MS) fragmentation (Figure 2C). Following correction for multiple testing, 93 Kla sites exhibited statistically significant differences between groups—61 were upregulated and 32 downregulated—with their distribution visualized via heat map (Figure 2D). Kyoto Encyclopedia of Genes and Genomes (KEGG) enrichment analysis revealed that proteins with differential lactylation were significantly enriched in energy metabolism pathways, including glycolysis and gluconeogenesis (Figure 2E).

Analysis at the pathway and network levels revealed that a protein–protein interaction (PPI) network constructed from proteins with significantly altered Kla sites positioned PGK1 at the center, underscoring its potential roles as the first ATP‐generating enzyme in glycolysis (Figure 2F). MS/MS spectral evidence further confirmed a notable change in lactylation of PGK1 at K361 in infected bone tissues (Figure 2G). Cross‐species sequence alignment showed that PGK1 K361 was highly conserved (Figure 2H), suggesting its functional relevance. Collectively, these findings suggest that during PTO‐associated metabolic reprogramming, regulation of glycolysis by lactylation may converge on PGK1 K361, offering a foundation for further mechanistic research and possible therapeutic strategies.

### 2.3 PGK1 K361 Lactylation Mediated Osteogenic Suppression Under Infection

**FIGURE 3:**
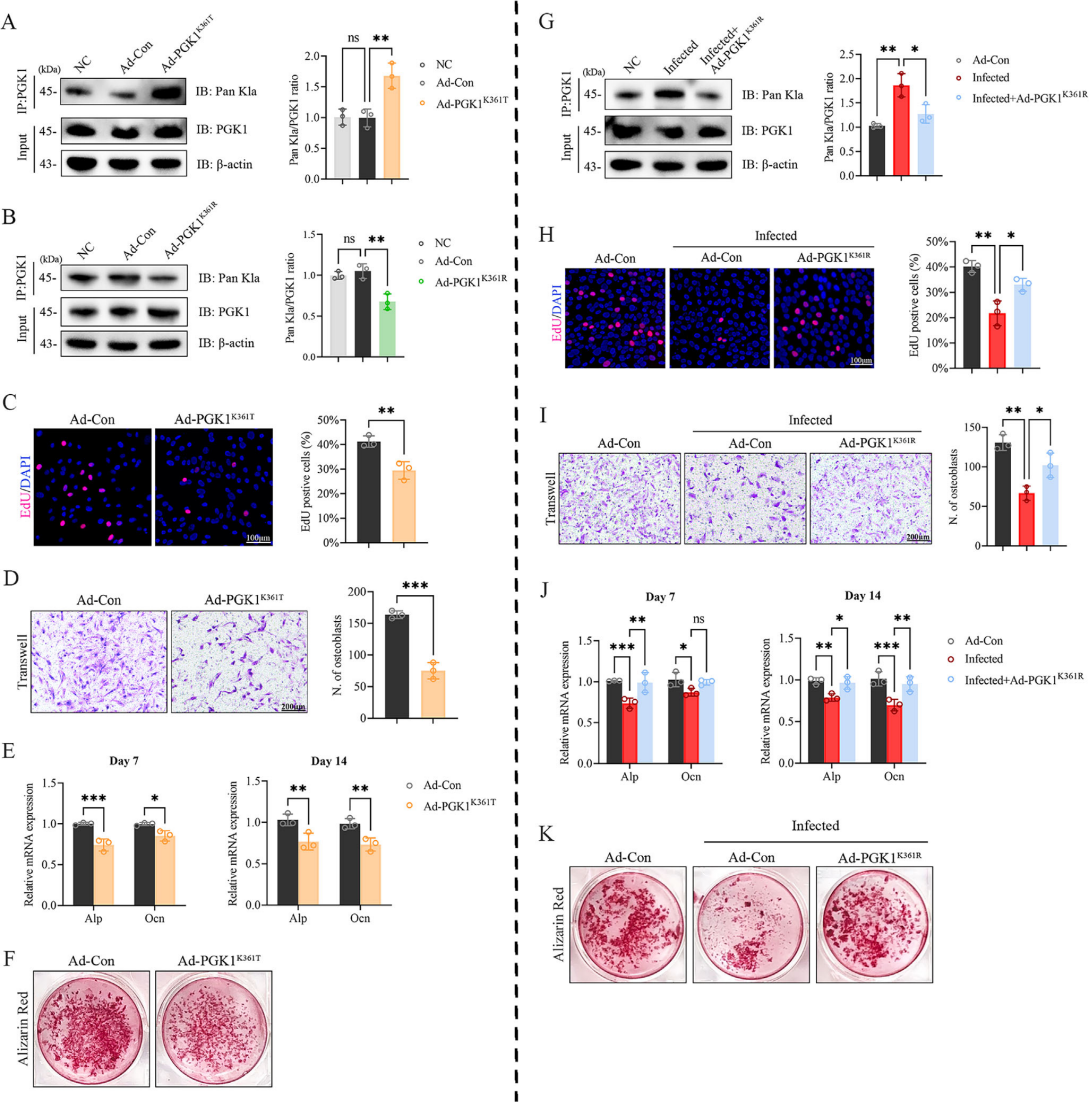
PGK1 K361 lactylation mediates osteogenic suppression under infection. (A,B) Immunoprecipitation/immunoblotting (IP‐IB) and quantification of PGK1 lactylation levels after Ad‐PGK1^K361T^ and Ad‐PGK1^K361R^ transfections. (C) Representative images and quantification of EdU after Ad‐PGK1^K361T^ transfection; Scale bar, 200 µm. (D) Representative images and quantification of the Transwell assay after Ad‐PGK1^K361T^ transfection; Scale bar, 100 µm. (E) RT‐qPCR analysis of *Alp* (day 7) and *Ocn* (day 14) mRNA levels during osteogenic induction following Ad‐PGK1^K361T^ transfection. (F) Representative images of Alizarin Red S staining on day 28 after Ad‐PGK1^K361T^ transfection. (G) IP‐IB and quantification of PGK1 lactylation following *S. aureus* infection and Ad‐PGK1^K361R^ treatment. (H) Representative images and quantification of EdU assay after *S. aureus* infection and Ad‐PGK1^K361R^ treatment; Scale bar, 200 µm. (I) Representative images and quantification of the Transwell assay after *S. aureus* infection and Ad‐PGK1^K361R^ treatment; Scale bar, 100 µm. (J) RT‐qPCR analysis of *Alp* (day 7) and *Ocn* (day 14) mRNA levels during osteogenic induction following *S. aureus* infection and Ad‐PGK1^K361R^ treatment. (K) Representative images of Alizarin Red S staining on day 28 after *S. aureus* infection and Ad‐PGK1^K361R^ treatment. Data represent mean ± SEM, *n* = 3 per group. Statistical significance was assessed using unpaired Student's *t*‐test (C–E) and one‐way ANOVA with Dunnett's multiple comparison test (A,B,G–J). ^*^ *p* < 0.05, ^**^ *p* < 0.01, ^***^ *p* < 0.001, ^****^ *p* < 0.0001, ns: no significance.

To elucidate the functional role of PGK1 K361, site‐specific adenoviral mutants were generated—Ad‐PGK1^K361T^ to mimic the lactylated form and Ad‐PGK1^K361R^ to represent the delactylated state. Immunoprecipitation (IP) analysis demonstrated increased and decreased PGK1 lactylation in the K361T and K361R mutants, respectively (Figure 3A,B). Functionally, the K361T variant markedly suppressed osteoblast proliferation and migration (Figure 3C,D), decreased *Alp* activity on day 7, suppressed *Ocn* expression on day 14 during osteogenic differentiation (Figure 3E), and reduced mineralized nodule formation on day 28 (Figure 3F).

Under *S. aureus* infection (MOI = 10), K361R markedly mitigated the impairments in cell proliferation and migration (Figure 3G–I). Additionally, *Alp* (day 7) and *Ocn* (day 14) mRNA expression was upregulated (Figure 3J), with mineralization restored on day 28 (Figure 3K). These data indicate that lactylation at K361 is sufficient to recapitulate the osteogenic suppression observed in high lactate and infection conditions, whereas delactylation at K361 confers a clear rescue effect in the infectious context.

### 2.4 PGK1 K361 Lactylation Induced Osteogenic Defects in PTO

**FIGURE 4:**
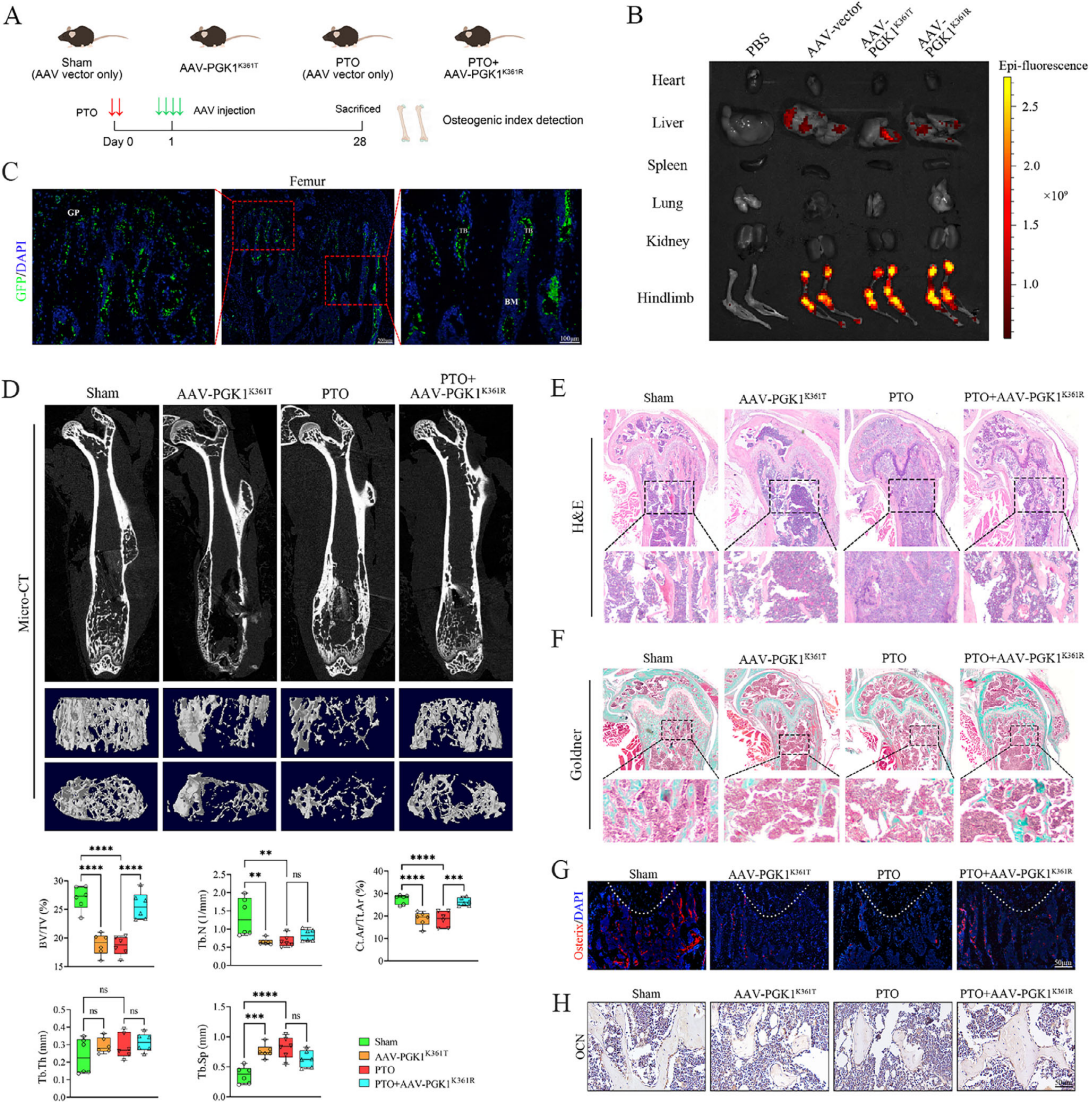
PGK1 K361 lactylation causes osteogenic defects in vivo. (A) Schematic of post‐traumatic osteomyelitis (PTO) mouse modeling and AAV administration timeline. (B) Individual organ imaging of green fluorescent protein (GFP) expression. (C) Representative immunofluorescence (IF) staining images of GFP in mouse femurs treated with AAV9‐GFP; Scale bar, 200 µm; magnified view, 100 µm. (D) Micro‐CT 3D reconstructions and quantitative parameters. BV/TV, bone volume/tissue volume; Tb.Th, trabecular thickness; Tb.Sp, trabecular separation; Tb.N, trabecular number; Ct.Ar/Tt.Ar, cortical area/total area. (E) Representative images of hematoxylin and eosin (H&E) staining; Scale bar, 200 µm; magnified view, 100 µm. (F) Representative images of Goldner's trichrome staining; Scale bar, 200 µm; magnified view, 100 µm. (G) Representative IF staining images of osterix in mouse femurs; white dotted line: growth plate; Scale bar, 50 µm. (H) Representative immunohistochemical (IHC) staining images of OCN in mouse femurs. Scale bar, 50 µm. Data represent mean ± SEM, *n* = 6 per group. Statistical significance was assessed using a one‐way ANOVA with Dunnett's multiple‐comparison test (D). ^*^ *p* < 0.05, ^**^ *p* < 0.01, ^***^ *p* < 0.001, ^****^ *p* < 0.0001, ns: no significance.

To extend the in vitro findings regarding the role of PGK1 K361 in determining osteogenic phenotypes, validation studies were performed in a murine PTO model. Following confirmation of effective osteoblast‐targeted delivery using rAAV9‐green fluorescent protein (GFP) under the control of the osterix (*Osx*) promoter (Figure S2A), AAV‐PGK1^K361T^ and AAV‐PGK1^K361R^ constructs were generated. The experimental workflow and dosing regimen are presented in Figure 4A.

Whole‐body and organ fluorescence imaging demonstrated predominant GFP expression in the hind limbs, with secondary expression observed in the liver and no detectable signals in the heart, spleen, lungs, or kidneys (Figure 4B). Immunofluorescence (IF) analysis of femoral tissue and other major organs confirmed this distribution pattern (Figure 4C; Figure S2B). Furthermore, hematoxylin and eosin (H&E) staining revealed no significant histopathological abnormalities in major organs (Figure S2C), indicating good tolerability.

Micro‐CT analysis revealed that both AAV‐PGK1^K361T^ and PTO decreased bone mass, whereas AAV‐PGK1^K361R^ markedly improved bone parameters. Specifically, bone volume/tissue volume (BV/TV) and cortical area/total area (Ct.Ar/Tt.Ar) increased, with upward trends in trabecular thickness (Tb.Th) and trabecular number (Tb.N) and a decrease in trabecular separation (Tb.Sp; Figure 4D). Histological evaluations supported these results; H&E staining demonstrated reduced trabecular area and number in the AAV‐PGK1^K361T^ and PTO groups, which were restored by AAV‐PGK1^K361R^ (Figure 4E). Goldner and Masson's trichrome staining showed more mineralized bone in the AAV‐PGK1^K361R^ group (Figure 4F; Figure S3A). K361 delactylation led to an increase in osteoblast numbers, whereas osteoclast counts remained unchanged (Figure 4G,H; Figure S3B–D). In vitro osteoclast differentiation assays (TRAP staining and osteoclast marker expression) also revealed no detectable differences with PGK1 K361 lactylation manipulation (Figure S4A,B).

Collectively, these findings indicate that PGK1 K361 lactylation status directly affects bone outcomes: lactylation drives bone loss, whereas inhibiting lactylation at this site selectively restores osteogenic function in PTO without altering bone resorption.

### 2.5 PGK1 K361 Acted Independently of Glycolytic Catalysis via Non‐Metabolic Signaling

Given that PGK1 is a key glycolytic enzyme, its catalytic activity was assessed. PGK1 enzymatic activity was not significantly altered by K361 lactylation (Figure S5A), and the transcript levels of glycolysis‐related genes did not differ significantly (Figure S5B). Seahorse assays further demonstrated consistent extracellular acidification profiles, with parameters such as glycolysis, glycolytic capacity, and glycolytic reserves remaining unaffected (Figure S5C). Additionally, mitochondrial oxygen consumption parameters were unchanged (Figure S5D). Collectively, these observations suggest that modification of PGK1 K361 does not alter glycolytic flux or oxidative phosphorylation (OXPHOS) function, but likely mediates effects through a non‐enzymatic, signaling‐based pathway.

**FIGURE 5:**
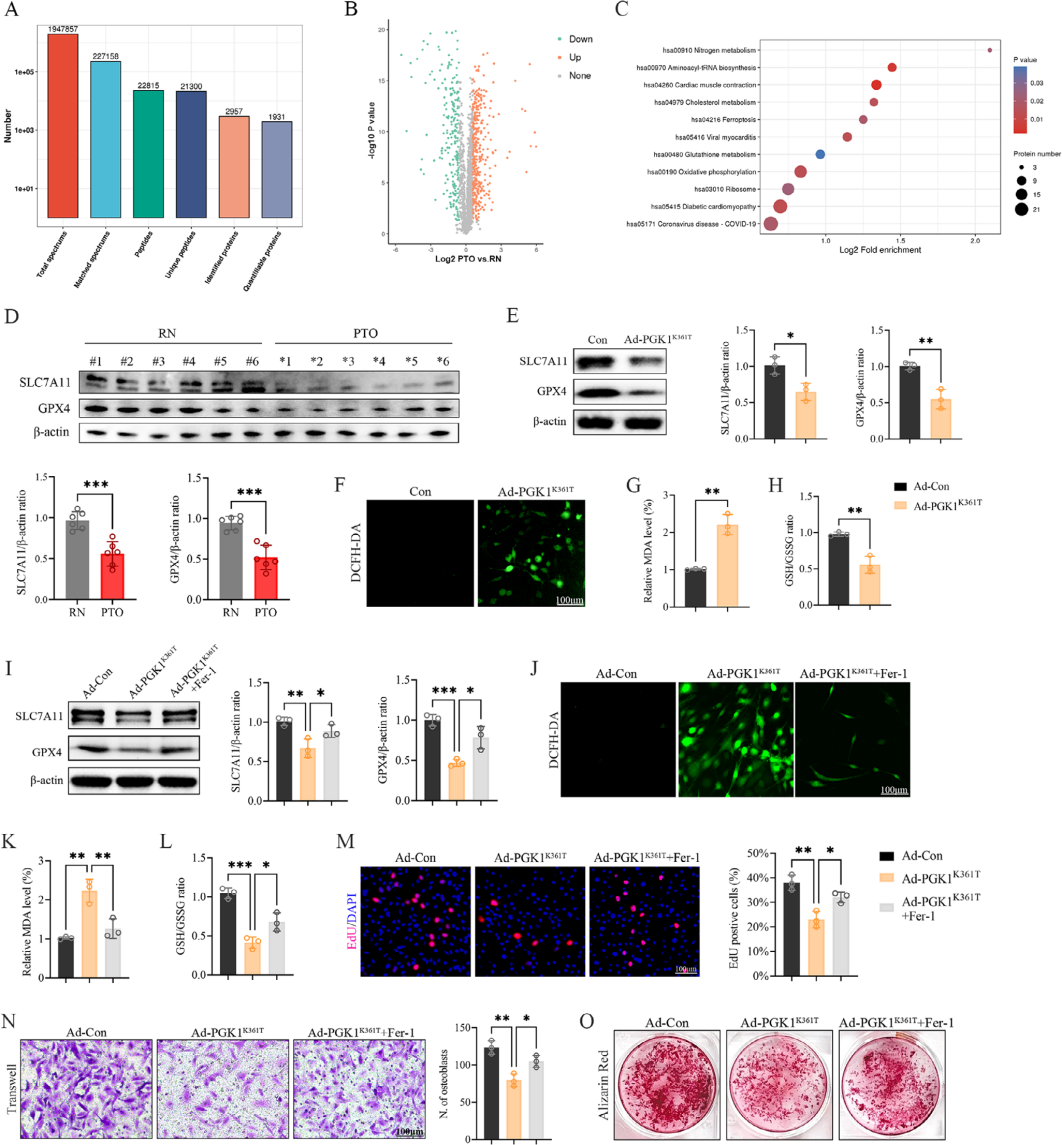
PGK1 K361 effects are independent of glycolytic catalysis and involve ferroptosis. (A) Overview of proteomic MS data and quality metrics. (B) Volcano plot of differentially expressed proteins. (C) KEGG pathway enrichment bubble plot. (D) Western blotting and quantification of ferroptosis markers in human bone tissues. (E) Western blot and quantification of ferroptosis markers after Ad‐PGK1^K361T^ transfection in osteoblasts. (F–H) Intracellular reactive oxygen species (ROS) and malondialdehyde (MDA) levels, and glutathione (GSH)/oxidized glutathione (GSSG) ratio changes after Ad‐PGK1^K361T^ transfection in osteoblasts. Scale bar, 100 µm. (I) Western blotting and quantification of ferroptosis markers after Ad‐PGK1^K361T^ transfection and Ferrostatin‐1 (Fer‐1) treatment of osteoblasts. (J–L) Intracellular ROS and MDA levels, and GSH/GSSG ratio changes after Ad‐PGK1^K361T^ transfection and Fer‐1 treatment of osteoblasts; Scale bar, 100 µm. (M–O) Effects of Fer‐1 on osteoblast proliferation (EdU), migration (Transwell), and mineralization (Alizarin Red S) after Ad‐PGK1^K361T^ transfection. Data represent mean ± SEM, *n* = 3 per group. Statistical significance was assessed using unpaired Student's *t*‐test (D, E, G, H) and one‐way ANOVA with Dunnett's multiple‐comparison test (I,K–N). ^*^ *p* < 0.05, ^**^ *p* < 0.01, ^***^ *p* < 0.001, ^****^ *p* < 0.0001, ns: no significance.

To further investigate potential mechanisms, whole‐proteomic profiling was performed on the same human bone tissue samples, yielding 2,957 proteins, of which 1,931 were not quantified (Figure 5A). Differential expression analysis identified 446 upregulated and 246 downregulated proteins (Figure 5B). KEGG enrichment analysis revealed a significant association with ferroptosis in the PTO group (Figure 5C). Notably, in human bone tissues, the PTO group exhibited significantly reduced levels of the ferroptosis markers glutathione peroxidase 4 (GPX4) and cystine/glutamate transporter (SLC7A11; Figure 5D).

Our previous research has demonstrated that *S. aureus* infection induces ferroptosis in osteoblasts, while administration of the ferroptosis inhibitor Ferrostatin‐1 (Fer‐1) restored impaired cellular function [16]. Building upon these findings, we specifically evaluated the ferroptosis phenotype and pharmacological intervention in the Ad‐PGK1^K361T^ model. After lactylation, the expression levels of GPX4 and SLC7A11 were downregulated (Figure 5E), accompanied by increased intracellular reactive oxygen species (ROS; Figure 5F), elevated malondialdehyde (MDA) levels (Figure 5G), and a reduced glutathione (GSH)/oxidized glutathione (GSSG) ratio (Figure 5H). Treatment with Fer‐1 effectively reversed these molecular and metabolic changes (Figure 5I–L), restoring osteoblast proliferation, migration, and osteogenic differentiation (Figure 5M–O). Collectively, these results suggest that lactylation at PGK1 K361 impairs osteogenic function primarily through the induction of ferroptosis rather than by modifying glycolytic catalysis and that inhibiting ferroptosis can rescue the observed phenotype.

### 2.6 VDAC3: A Selective PGK1 K361 Lactylation Downstream Hub Driving Mitochondrial Ferroptosis

**FIGURE 6:**
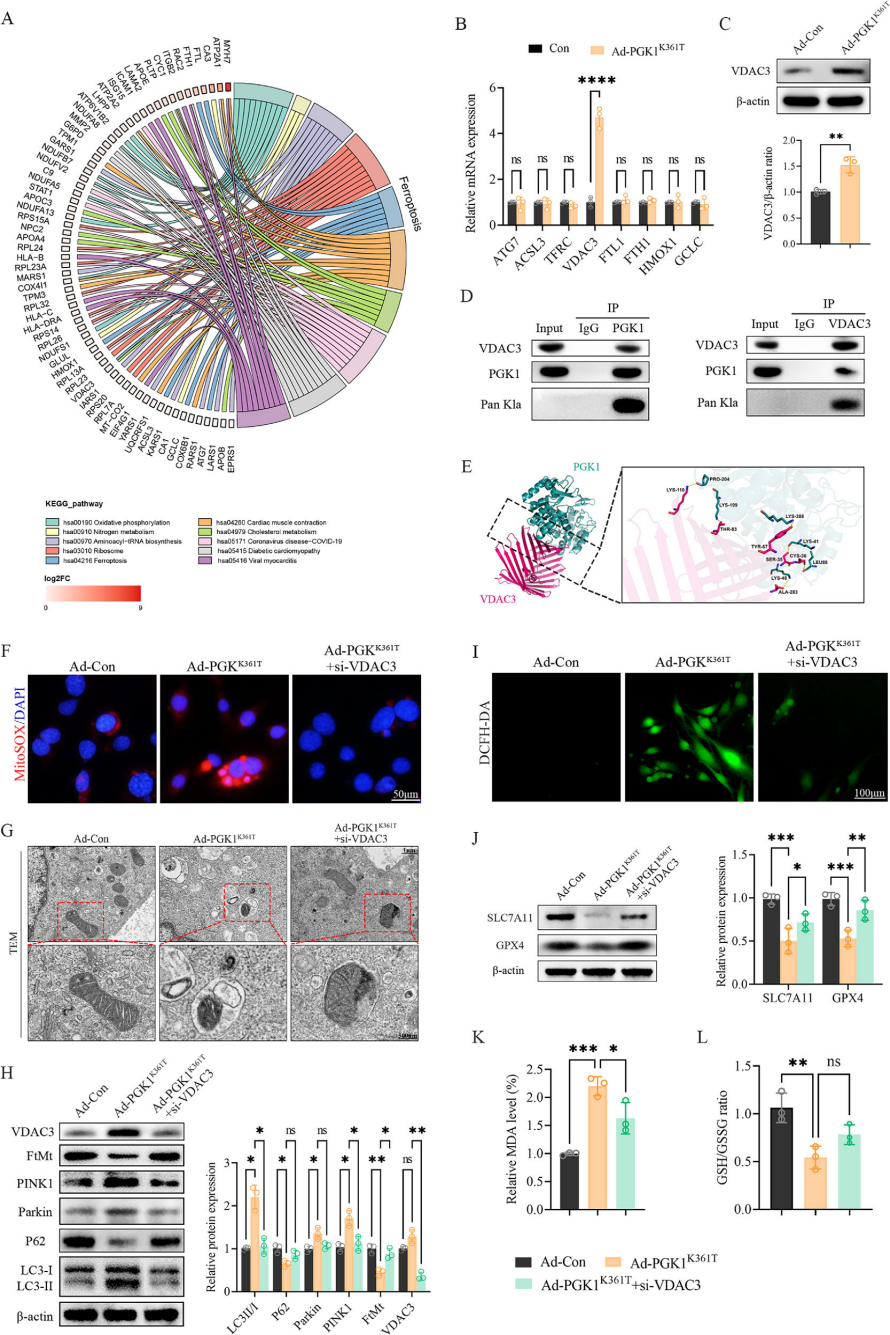
VDAC3 is a selective downstream hub of PGK1 K361 (Kla) mediating a mitophagy–ferroptosis axis. (A) Chord diagram of upregulated differentially expressed proteins (DEPs) from patient bone proteomes. (B) Validation of ferroptosis‐related candidates by RT‐qPCR after Ad‐PGK1^K361T^ transfection. (C) Western blotting and quantification of VDAC3 after Ad‐PGK1^K361T^ transfection. (D) Co‐immunoprecipitation (Co‐IP) of the interaction between PGK1 K361 (Kla) and VDAC3. (E) Molecular docking supporting a stable binding interface between PGK1 and VDAC3. (F) Representative images of MitoSOX cells after Ad‐PGK1^K361T^ and si‐VDAC3 transfection; Scale bar, 50 µm. (G) Representative transmission electron microscopy (TEM) images of osteoblasts after Ad‐PGK1^K361T^ and si‐VDAC3 transfection; Scale bar, 1 µm; magnified view, 500 nm. (H) Western blotting and quantification of mitophagy‐related proteins after Ad‐PGK1^K361T^ and si‐VDAC3 transfection. LC3II was quantified as the LC3II/LC3I ratio; P62, Parkin, PINK1, FtMt, and VDAC3 were normalized to *β*‐actin. (I) Representative images of intracellular ROS levels after Ad‐PGK1^K361T^ and si‐VDAC3 transfection; Scale bar, 100 µm. (J–L) Effects of VDAC3 knockdown on ferroptosis phenotypes: western blotting and quantification of ferroptosis markers, MDA levels, and GSH/GSSG ratio. FtMt: Mitochondrial Ferritin. Data represent mean ± SEM, *n* = 3 per group. Statistical significance was assessed using the unpaired Student's *t*‐test (A,B,H) and one‐way ANOVA with Dunnett's multiple‐comparison test (I,J,M–O). ^*^ *p* < 0.05, ^**^ *p* < 0.01, ^***^ *p* < 0.001, ^****^ *p* < 0.0001, ns: no significance.

A chord diagram illustrating the upregulated differentially expressed proteins (DEPs) in patient bone proteomes identified several ferroptosis‐related changes in autophagy‐related 7 (ATG7), acyl‐CoA synthetase long‐chain family member 3 (ACSL3), voltage‐dependent anion‐selective channel protein 3 (VDAC3), ferritin light chain 1 (FTL1), ferritin heavy chain 1 (FTH1), heme oxygenase 1 (HMOX1), and glutamate–cysteine ligase (GCLC) in the PTO group (Figure 6A). To identify key downstream nodes, qPCR validation was performed for these candidates, including transferrin receptor 1 (TFRC)—a downregulated DEP associated with ferroptosis. VDAC3 showed increased transcription levels, consistent with the proteomic trend, which reached statistical significance (Figure 6B). Western blotting analysis further verified the protein‐level upregulation of VDAC3 (Figure 6C), suggesting that VDAC3 was selectively upregulated rather than global activation of the ferroptosis pathway.

Co‐immunoprecipitation (Co‐IP) analyses demonstrated physical interactions between PGK1 K361 (Kla) and VDAC3 (Figure 6D). Additionally, molecular docking analysis revealed a stable binding interface in a specific region (Figure 6E), supporting the structural basis for this experimental interaction.

Functionally, Ad‐PGK1^K361T^ induced canonical markers of mitochondrial stress and a ferroptotic phenotype in osteoblasts, as evidenced by decreased mitochondrial membrane potential (MMP; Figure S6A), increased mitochondrial ROS and LC3 expression (Figure S6B,C), and alterations in mitophagy markers (Figure S6D). Notably, mitochondrial ferritin (FtMt)—a mitochondria‐localized iron‐storage protein responsible for sequestering Fe^3^^+^—plays a critical role in regulating the labile Fe^2^^+^ pool required for Fenton chemistry and mitigating ROS generation. These findings suggest activation of the VDAC3–FtMt–PINK1/Parkin‐dependent mitophagy–ferroptosis axis.

To establish causality, a si‐VDAC3 was generated and validated (Figure S6E). VDAC3 knockdown attenuated the mitophagy and ferroptosis phenotypes, as demonstrated by reductions in both mitochondrial and intracellular ROS levels, improved mitochondrial ultrastructure, normalization of mitophagy and ferroptosis biomarkers, restoration of MDA concentrations, and recovery of the GSH/GSSG ratio (Figure 6F–L). Collectively, these findings support a mechanistic model in which lactylation at PGK1 K361, via binding to and upregulation of VDAC3, disrupts mitochondrial iron homeostasis, promotes excessive mitophagy, and ultimately induces ferroptosis, thereby suppressing osteoblast function.

### 2.7 Delactylation of PGK1 K361 Prevented Ferroptosis by Restoring Mitochondrial Homeostasis

**FIGURE 7:**
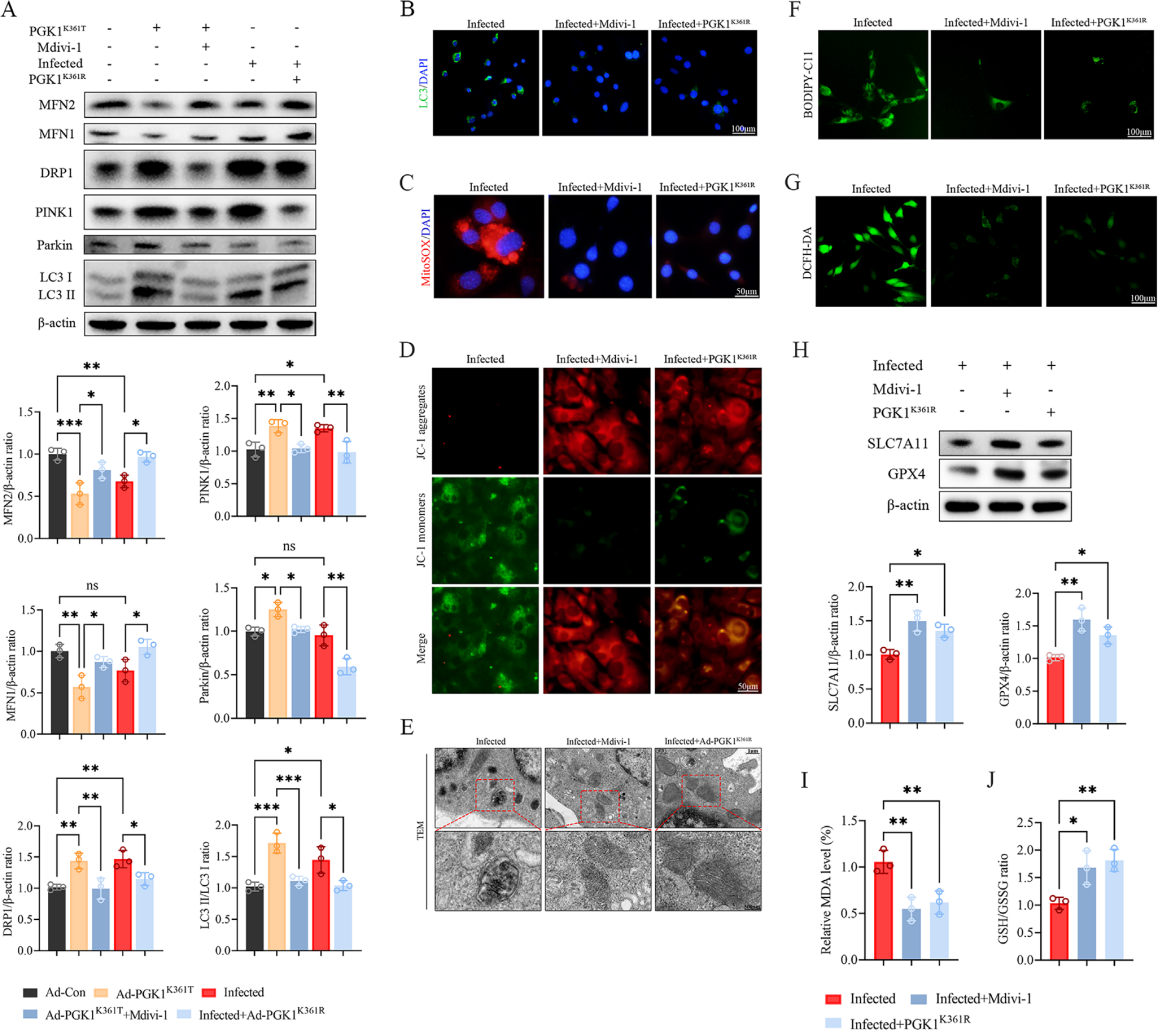
Delactylation of PGK1 K361 restores mitochondrial homeostasis and prevents ferroptosis. (A) Western blotting and quantification of mitophagy and mitochondrial dynamic markers showing the effects of Mdivi‐1 and Ad‐PGK1^K361R^. (B) Representative images of LC3 ICC; Scale bar, 100 µm. (C,D) Mitochondrial ROS (MitoSOX) and mitochondrial membrane potential (JC‐1) following treatment with Mdivi‐1 and Ad‐PGK1^K361R^ interventions; Scale bar, 50 µm. (E) Representative TEM images of osteoblasts following interventions; Scale bar, 1 µm; magnified view, 500 nm. (F,G) Lipid and intracellular ROS levels after Mdivi‐1 and Ad‐PGK1^K361R^ treatment; Scale bar, 100 µm. (H–J) Effects of Mdivi‐1 and Ad‐PGK1^K361R^ treatment on ferroptosis phenotypes: western blotting and quantification of ferroptosis markers, MDA levels, and GSH/GSSG ratio. Data represent mean ± SEM, *n* = 3 per group. Statistical significance was assessed using a one‐way ANOVA with Dunnett's multiple‐comparison test (A,G–I). ^*^ *p* < 0.05, ^**^ *p* < 0.01, ^***^ *p* < 0.001, ^****^ *p* < 0.0001, ns: no significance.

As PGK1 K361 (Kla) has been shown to simultaneously induce mitochondrial damage and ferroptosis, the mitochondrial fission inhibitor Mdivi‐1 was employed to stabilize mitochondrial homeostasis. Immunoblotting (IB) analysis demonstrated that under Ad‐PGK1^K361T^ infection conditions, Mdivi‐1 attenuated markers of mitophagy and fission while enhancing fusion markers. Similarly, Ad‐PGK1^K361R^ led to comparable changes (Figure 7A). LC3 ICC confirmed that both Mdivi‐1 and Ad‐PGK1^K361R^ effectively suppressed mitophagy (Figure 7B). Functional assays utilizing MitoSOX and JC‐1 indicated that Ad‐PGK1^K361R^ decreased mitochondrial ROS, restored MMP, and improved mitochondrial ultrastructure (Figure 7C–E), with concomitant reductions in lipid ROS and intracellular ROS levels (Figure 7F,G). Subsequent evaluation of ferroptosis markers revealed that both interventions upregulated GPX4 and SLC7A11 expression (Figure 7H), while decreasing MDA levels and increasing the GSH/GSSG ratio (Figure 7I,J). Cumulatively, these findings suggest that lactylation at PGK1 K361 compromises mitochondrial homeostasis by driving mitophagy and triggering ferroptosis. In contrast, delactylation at this residue disrupts this pathological axis and restores osteoblast function.

## 3 Discussion

In this study, we identified a previously unrecognized mechanism by which lactate accumulation during bone infection promotes osteogenic activity via protein lysine lactylation. Analyses of bone tissue from patients with PTO and experimental infection models revealed increased lactate levels, accompanied by a global increase in protein lactylation. Functional assays demonstrated that a lactylation‐mimetic mutation at PGK1 K361 markedly impaired osteoblast proliferation, migration, and differentiation, leading to reduced bone mass in mouse models. Conversely, a delactylation mimic effectively reversed infection‐induced deficits in osteogenesis. Collectively, these results highlight lactylation of PGK1 at K361 as a critical molecular event underlying infection‐driven loss of osteogenic function.

Considering that bone loss during infection is governed by the interplay between bone formation and resorption [17], we further investigated whether PGK1 K361 lactylation influences osteoclastogenesis and osteoclast abundance. Emerging evidence suggests that lactylation can directly affect osteoclast programming in specific contexts; for instance, histone lactylation reportedly suppresses NFATc1‐mediated osteoclastogenesis [18, 19]. To assess this in our system, we performed additional in vitro osteoclast differentiation assays (Figure S4A,B) and found that manipulating PGK1 K361 lactylation did not alter TRAP‐positive multinucleated osteoclast formation or the expression of canonical osteoclastogenic markers (NFATc1, c‐Fos, c‐Src, CTSK, and MMP9). In vivo TRAP staining across mouse groups also showed no significant differences in osteoclast abundance or distribution, and quantification confirmed stable osteoclast numbers (Figure S3B,C). These findings support the conclusion that suppression of PGK1 K361 lactylation confers bone‐protective effects primarily through osteoblast‐intrinsic mechanisms, rather than via altered osteoclast‐mediated bone resorption. In this context, we investigated the deleterious impact of PGK1 K361 lactylation on osteoblasts.

Notably, lactylation of PGK1 at K361 did not affect its glycolytic activity or the transcription of glycolysis‐associated genes, as evidenced by Seahorse XF analysis, which demonstrated unaltered extracellular acidification rate (ECAR)‐based glycolytic function (Figure S5). Instead, it acts through non‐enzymatic functions. Proteomic analyses and validation experiments suggested that PGK1 K361 lactylation selectively upregulated VDAC3 and enhanced PGK1–VDAC3 interactions. Voltage‐dependent anion channels (VDACs) in the outer mitochondrial membrane regulate metabolic flux and serve as signaling platforms [20]. While VDAC1 has been extensively characterized [21], the functions of VDAC3 remain less well defined but have been associated with mitochondrial homeostasis, redox regulation, and ferroptosis [22]. In the infectious bone milieu, mitochondria are inevitably exposed to oxidative stress and damage [23]. Mitophagy, primarily via the PINK1–Parkin pathway [24], is essential for removing damaged mitochondria. Although moderate mitophagy is protective, excessive activation can deplete mitochondrial mass, precipitating an energy crisis and cell death [25]. Recent research into musculoskeletal degeneration reinforces the notion that mitochondrial quality control intersects with inflammatory cell death pathways. For example, targeting PTPN22 in intervertebral disc degeneration modulates mitophagy and mitigates pyroptosis‐related pathology via the PI3K–AKT–mTOR axis [26]. Pharmacological enhancement of mitophagy mitigates degenerative damage and alleviates intervertebral disc degeneration by activating the NRF2–PINK1 pathway, suggesting that boosting mitophagy may be protective in stressed musculoskeletal tissues [27]. Our data showed that VDAC3 was excessively activated under infection conditions, resulting in decreased MMP, increased mitochondrial ROS, reduced FtMt expression, and robust activation of the PINK1–Parkin pathway, collectively driving overactive mitophagy and ferroptosis.

Ferroptosis is an iron‐dependent, lipid peroxidation‐driven, non‐apoptotic mode of cell death increasingly recognized in inflammation‐ and infection‐associated bone cells [28]. Key determinants include the labile iron pool, GPX4, polyunsaturated lipid substrates, and mitochondrial metabolism [29]. Our proteomic profiling of clinical specimens identified significant changes in several ferroptosis‐related proteins in infected bone tissues. Dysregulation of proteins involved in iron uptake and storage (such as TFRC and FTL/FTH1) may expand the labile iron pool and increase redox‐active Fe^2^^+^, facilitating Fenton reactions and lipid peroxidation [30]. Stress‐responsive redox regulators, such as HMOX1 and GCLC, were also altered; HMOX1 increases intracellular free iron via heme turnover under inflammatory stress, whereas GCLC determines GSH biosynthetic capacity, thereby impacting GPX4‐dependent detoxification of lipid hydroperoxides [31, 32]. Additionally, changes in proteins associated with lipid metabolism and autophagy (for example, ACSL family members and ATG7) could further influence ferroptosis susceptibility by modulating membrane phospholipid profiles and adaptive‐stress responses [33].

Canonical ferroptosis regulators, such as ACSL4, which promotes the enrichment of peroxidation‐prone polyunsaturated fatty acid‐containing phospholipids, and NCOA4, which drives ferritinophagy and iron release, are widely implicated in ferroptotic signaling in various contexts [34]. However, in our patient‐derived proteomics data, under the current detection depth and statistical thresholds, we did not observe significant changes in ACSL4 and NCOA4 protein levels. This suggests that the pathways regulating ferroptosis in infected bone tissues may vary depending on disease stage, cell type, and local microenvironmental factors and may involve post‐translational or compartment‐specific controls not detectable by bulk proteomics methods.

To further correlate our clinical findings with specific mechanisms, we investigated which ferroptosis‐related proteins, altered in patient samples, directly respond to PGK1 K361 lactylation. By modifying PGK1 K361 lactylation in osteoblasts, we found that VDAC3 was consistently and robustly regulated, while other candidate proteins showed no similar patterns. Through additional interaction and loss‐of‐function experiments, our results suggest that VDAC3 serves as a key downstream target of PGK1 K361 lactylation, linking lactylation changes within the bone infection microenvironment to FtMt/PINK1/Parkin‐mediated mitophagy and osteoblast ferroptosis. FtMt is essential for maintaining iron homeostasis and reducing lipid peroxidation [35]. In an infectious setting, disruptions in FtMt, along with excessive mitophagy, can subject osteoblasts to compounded metabolic stress and increased susceptibility to ferroptosis [36, 37]. We postulate that lactylation acts as a form of metabolic “memory,” translating infection‐related lactate accumulation into PTM signals. PGK1 K361 lactylation acts as a molecular switch, altering protein interactions and slowing mitochondrial quality control. As a downstream mediator, VDAC3 integrates signals from iron flux, ROS production, and PINK1/Parkin‐mediated mitophagy, ultimately promoting ferroptosis. This mechanism helps explain the clinical observation that bone mass and osteogenic capacity often do not fully recover even after infection appears resolved—lactylation may epigenetically lock osteoblasts into a vulnerable state.

Using clinical samples, we established a causal chain from infection‐induced lactate accumulation to protein lactylation and, ultimately, to impaired bone formation. Moreover, lactylation endows the glycolytic enzyme PGK1 with regulatory roles beyond metabolism, underscoring the diverse functions of PTMs in metabolic enzymes involved in cell fate determination. Thus, PGK1 K361 lactylation may serve as a molecular marker of osteogenic impairment in bone infections. Additionally, silencing P300—a key enzyme mediating lactylation—reduced lactylation levels and restored osteogenic function, identifying lactylation machinery as a potential therapeutic target. Our data also identified VDAC3 as the central node for the lactylation–ferroptosis pathway, expanding insights into the crosstalk between mitochondrial homeostasis and iron metabolism. Importantly, inhibiting VDAC3 inhibits ferroptosis while preserving mitochondrial stability. These findings suggest that locally delivering delactylation mimetics, VDAC3 inhibitors, or FtMt‐stabilizing interventions combined with antimicrobial therapy may offer integrated approaches to combat infection while preserving bone regeneration.

## 4 Conclusion

In summary, this study demonstrates that PGK1 K361 lactylation, via the VDAC3–FtMt–PINK1/Parkin axis, drives mitophagy and ferroptosis within the infected bone microenvironment, ultimately impairing osteogenesis. This mechanism expands the understanding of lactylation's biological roles in bone infections and identifies actionable molecular targets and potential strategies for clinical intervention.

## 5 Materials and Methods

### 5.1 Human Bone Samples

This study was approved by the Ethics Committee of Nanfang Hospital, with all procedures conducted in accordance with the principles outlined in the 1964 Declaration of Helsinki (Approval Number: NFEC‐2022‐194). Human bone specimens were obtained from patients undergoing debridement surgery, with informed consent.

Inclusion in the PTO group required a confirmed diagnosis of osteomyelitis via either a positive tissue bacterial culture or histological examination. Bone tissue from patients with non‐infected fractures served as controls. Both groups met the following inclusion criteria: (a) age between 18 and 55 years with no history of osteoporosis; (b) consent to undergo surgery; and (c) absence of systemic disease or immune dysfunction (e.g., diabetes, metabolic bone disease, malignant tumors, or severe hepatic and renal dysfunction). A total of 14 bone samples were collected, comprising six controls and eight PTO cases. The bone tissue was processed into powder using a cryogenic grinder (Luka Sequencing Instrument Co. Ltd., Guangzhou, China) and stored at −80°C for subsequent use.

### 5.2 Quantitative Lactylproteomics Analysis

Human bone tissues (RN, relative normal, *n* = 6; PTO, n = 8) were cryogenically ground in liquid nitrogen and lysed (4× volume; 1% SDS, protease inhibitor cocktail, 3 µm TSA, 50 mm NAM). After sonication, the lysates were centrifuged at 4°C and 12,000 × g for 10 min, and proteins were quantified using a BCA assay. Equal protein quantities were precipitated with 20% TCA at 4°C for 2 h, collected by centrifugation at 4500 × g for 5 min, washed 2–3 times with cold acetone, redissolved in 200 mm TEAB, reduced (5 mm DTT, 56°C, 30 min), alkylated (11 mm IAM, room temperature, dark, 15 min), and digested overnight with trypsin (1:50, w/w).

Peptides were dissolved in IP buffer (100 mm NaCl, 1 mm EDTA, 50 mm Tris‐HCl, 0.5% NP‐40, pH 8.0) and enriched overnight at 4°C using an anti‐lactyl‐lysine resin (PTM‐1404, PTM Bio, Hangzhou, China). The resin was then washed four times with IP buffer and twice with water, peptides were eluted with 0.1% TFA three times, vacuum‐dried, desalted with C18 ZipTips, and dried again for LC–MS/MS.

The peptides were separated with a NanoElute UHPLC system (450 nL min^−^^1^ flow rate) using solvents A(0.1% FA in water with 2% can) and B (0.1% FA in ACN) over a gradient: 7%–24% B (0–42 min), 24%–32% B (42–54 min), 32%–80% B (54–57 min), and 80% B (57–60 min). Separation was followed by analysis on a timsTOF Pro in PASEF mode (capillary voltage 1.6 kV; m/z range 100–1700; 10 PASEF scans per cycle; dynamic exclusion 24 s). Raw data files were searched against a study‐specific protein database and underwent quality control at the peptide and PTM site levels. Further analyses included functional annotation (GO, KEGG, Protein domain, COG/KOG, and STRING), quantification and reproducibility assessment at the site level, differential analysis with visualization, motif analysis of regulated sites, and functional classification (GO level‐2, subcellular localization, and COG/KOG).

### 5.3 Bacterial Strains and Culture

*S. aureus* (ATCC 6538) was used in this study. Single bacterial colonies were cultured overnight in tryptic soy broth on a rotary shaker at 150 rpm and 37°C, followed by dilution to the required concentration before use.

### 5.4 Cell Culture and Treatment

Murine pre‐osteoblast MC3T3‐E1 cells served as osteoblasts in in vitro assays. Cells were cultured in Minimum Essential Medium Alpha (*α*‐MEM, Gibco, Thermo Fisher Scientific, Waltham, MA, USA) with 10% fetal bovine serum and 100 U mL^−1^ penicillin/streptomycin solution at 37°C in a 5% CO_2_ environment. Cells were osteogenically induced by treating with 50 µg mL^−1^ ascorbic acid and 2 mm *β*‐glycerol phosphate for 7, 14, or 28 days.

To induce intracellular infection, MC3T3‐E1 cells were exposed to *S. aureus* at various MOIs; MOI 10 was deemed optimal and used for subsequent experiments. For NALA treatments, a concentration gradient was established based on previous studies [16]. Fer‐1 and Mdivi‐1 were added to cell cultures 1 h before transfection.

### 5.5 Adenoviral Transduction and siRNA Transfection

Replication‐deficient adenoviruses expressing site‐specific PGK1 mutations were used. Lysine (K) at position 361 of PGK1 was mutated to threonine (T) to mimic the lactylated state or with arginine (R) to simulate the non‐lactylated form, generating Ad‐PGK1^K361T^ and Ad‐PGK1^K361R^ constructs, respectively. The adenoviruses were designed and synthesized by Hanbio Co., Ltd. (Shanghai, China). The half‐volume infection method was used according to the manufacturer's protocol: during viral infection, half the volume of fresh culture medium was added to the adenovirus solution. After 4 h, the remaining medium was added to reach the full culture volume. Cells were harvested 72 h post‐infection to evaluate transduction efficiency by western blot analysis and for subsequent experiments.

Small interfering RNAs (siRNAs) targeting p300 and VDAC3, as well as control siRNAs, were synthesized by Hanbio Co., Ltd. (Shanghai, China). Primer sequences are provided in Table S2. After reaching ∼80%–90% confluency, osteoblasts were transfected with siRNAs using RNAfit (Hanbio Co. Ltd., Shanghai, China) according to the manufacturer's instructions.

### 5.6 Osteoblasts Function Assay

#### 5.6.1 Cell Viability Assay

The Cell Counting Kit‐8 (CCK‐8; Dojindo Laboratories, Kumamoto, Japan) was employed for assessment. Cells were treated according to the experimental design and incubated for 0, 24, 48, or 72 h. Subsequently, the CCK‐8 reagent was added and incubated for 30 min. The optical density at 450 nm (OD450) was measured using a microplate reader (Spark, Tecan Group Ltd., Männedorf, Switzerland).

#### 5.6.2 Cell Proliferation Assay

Cell proliferation was assessed using a YF@555 Click‐iT EdU Imaging Kit (UElandy, Suzhou, China). Following treatment, cells were incubated with 10 µm EdU reagent for 2 h. Subsequently, cells were fixed, permeabilized, and stained with EdU. After counterstaining with Hoechst 33342, the proportion of cells incorporating EdU was evaluated using an inverted fluorescence microscope (Leica Dmi8 + DFC, Wetzlar, Germany).

### 5.7 Osteogenic Ability Detection

Osteoblast mineralization was assessed using Alizarin Red S staining (A5533, Sigma–Aldrich, St. Louis, MO, USA) according to the manufacturer's protocol. Calcium deposits, indicated by red areas, were visualized under a light microscope (OLYMPUS BX53, Tokyo, Japan).

### 5.8 Measurement of Lactate Levels

Lactate concentrations in human bone tissue and cell medium were measured using a Lactic Acid Assay Kit (A019‐2‐1; Nanjing Jiancheng Bioengineering Institute, Nanjing, China), according to the manufacturer's instructions.

### 5.9 Immunoprecipitation (IP), Co‐Immunoprecipitation (Co‐IP) and Western Blotting

Cells were lysed on ice for 30 min in NP‐40 buffer (50 mm Tris‐HCl, pH 7.4, 150 mm NaCl, 1% NP‐40, 1 mm EDTA) supplemented with protease/phosphatase inhibitors; for PTM‐preserving assays, 50 mm NAM, 3 µm TSA, and 50 µm PR‐619 were also included. Lysates were cleared (12,000 × g, 10 min, 4°C), and protein concentration was quantified using the BCA assay. For IP, 0.5–1.0 mg total protein was pre‐cleared with control IgG plus protein A/G beads (4°C, 1 h), followed by overnight incubation with primary antibodies at 4°C and subsequent incubation with protein A/G beads (30 µL slurry, 2 h, 4°C). Beads were washed with lysis buffer, and bound proteins were eluted by boiling in 2× SDS sample buffer. The same procedure was applied for Co‐IPs under non‐denaturing conditions, including reciprocal Co‐IPs. Input (10% lysate), specific IP, and species‐matched IgG IP served as controls.

Protein samples were resolved using SDS‐PAGE and transferred onto polyvinylidene difluoride or nitrocellulose (NC) membranes. Membranes were blocked with Protein Free Rapid Block buffer (PS108P, EpiZyme), incubated with primary antibodies overnight at 4°C, and probed with horseradish peroxidase‐conjugated secondary antibodies. Signals were detected utilizing ECL and captured with a chemiluminescence system (ChemiScope 6100Touch, CLINX, China). Band intensities were quantified using ImageJ software, normalized to housekeeping proteins (*β*‐actin) or input for IP/Co‐IP, and expressed relative to the control.

### 5.10 Quantitative Real‐Time Polymerase Chain Reaction (qRT‐PCR)

Total RNA was extracted with TRIzol reagent and reverse‐transcribed into cDNA using a reverse transcription kit (AG11706, AGbio, Hunan, China). qRT‐PCR was performed on a real‐time fluorescence quantitative PCR instrument (ABI, Thermo Fisher Scientific, Waltham, MA, USA). Amplification differences between samples were calculated based on the 2^−ΔΔCt^ method and normalized to *GAPDH*. Primer sequences are detailed in Table S3.

### 5.11 PGK1 Activity Assay

PGK1 activity was measured using a Phosphoglycerate Kinase Activity Assay Kit (ab252890, Abcam, Cambridge, UK) according to the manufacturer's instructions. Optical density at 340 nm was recorded at 37°C in kinetic mode using a microplate reader (Spark, Tecan Group Ltd., Männedorf, Switzerland).

### 5.12 Measurement of ECAR

The ECAR and oxygen consumption rate (OCR) were measured using the Agilent Seahorse XFe96 Extracellular Flux Analyzer (Agilent Technologies, Santa Clara, CA, USA) with a Seahorse XF Cell Glycolysis Stress Test Kit (103020‐100) and Seahorse XF Cell Mito Stress Test Kit (103015‐100). Osteoblasts were washed with XF medium and incubated for 1 h in a CO_2_‐free incubator at 37°C before loading.

For ECAR (mpH min^−1^) measurements, glucose (20 mm), oligomycin (1 µm), and 2‐deoxy‐glucose (2‐DG, 20 mm) were assessed. OCR (pmol min^−1^) was measured using oligomycin (1.5 µm), FCCP (1.5 µm), antimycin A (0.5 µm), and rotenone (0.5 µm). All data were normalized to cell numbers.

### 5.13 Transmission Electron Microscopy (TEM)

Osteoblasts were seeded in 6‐well plates and cultured as previously described. Following treatment, cells were fixed in 2.5% glutaraldehyde for 5 min. Subsequently, the cells were carefully collected in the same fixative solution, and TEM images were captured (Hitachi, Ltd., Tokyo, Japan).

### 5.14 Animals

All animal experiments were conducted in accordance with protocols approved by the Animal Ethics Committee of Shenzhen People's Hospital (Approval Number: AUP‐230725‐THJ‐0281‐01). Twenty‐four male C57BL/6J mice, aged 12 weeks, were purchased from our hospital's Laboratory Animal Center and randomly divided into four groups (*n* = 6/group): Sham, AAV‐PGK1^K361T^, PTO, and PTO + AAV‐PGK1^K361R^.

To establish the PTO animal model, a single cortical defect was created using a 27‐gauge syringe needle positioned 2 mm proximal to the femoral condyle. In PTO mice, 2 µL of *S. aureus* solution at 1 × 10^6^ colony forming units mL^−1^ was slowly injected into the bone marrow cavity with a microsyringe. For the Sham and AAV‐PGK1^K361T^ groups, an equivalent volume of sterile PBS was injected. The drilled hole was then sealed with bone wax, and the incision was closed with a 4‐0 suture. One month post‐surgery, all mice were euthanized, and their femurs were collected for further analysis.

### 5.15 Adeno‐Associated Virus‐9 (AAV9) Construction and Injection

AAV9 was used as the vector to express site‐specific PGK1 mutants in mice. The AAV9 vector was modified with the pre‐osteoblast‐specific *Osx* promoter to ensure exclusive targeting of osteoblast lineage cells; the vector was designed and synthesized by WZ Biosciences, Inc. (Shangdong, China). Each mouse received 4 × 10^11^ GC AAV9 diluted with PBS to a total volume of 100 µL via tail vein injection following PTO surgery. Control mice received 100 µL PBS through the tail vein. Four weeks post‐injection, tissues were harvested for imaging of GFP expression in individual organs.

### 5.16 Micro‐CT

The harvested femur specimens were fixed in 4% paraformaldehyde for 36 h and scanned with a Micro‐CT (VivaCT80; Scanco Medical AG, Bassersdorf, Switzerland) at 55 kV, 145 µA, 8 W, and 10.4 µm resolution. The BV/TV, Tb.Th, Tb.Sp, Tb.N, and Ct.Ar/Tt.Ar was analyzed.

### 5.17 Histology and Immunohistochemistry (IHC)

Mouse femurs were decalcified in 0.5 m ethylenediaminetetraacetic acid (pH 8.0) for 2 weeks after micro‐CT scanning and embedded in paraffin. Specimens were coronally sectioned at 4‐µm‐thickness for H&E, Goldner trichrome (G3550, Solarbio, Beijing, China), Modified Masson's trichrome (G1346, Solarbio, Beijing, China), and TRAP (G1492, Solarbio, Beijing, China) staining. IHC staining was performed with an osteocalcin antibody (23418‐1‐AP, Proteintech Group, Inc., Rosemont, IL, USA) and visualized using a light microscope (OLYMPUS BX53, Tokyo, Japan).

### 5.18 Immunocytochemistry (ICC) and IF

Cells were fixed, permeabilized, and blocked with 5% BSA. For IF staining, deparaffinized sections were also blocked with 5% BSA. Samples were incubated overnight at 4°C with various primary antibodies, followed by incubation with the corresponding secondary antibody (Dylight 488/594, Abbkine Scientific Co., Ltd., Atlanta, GA, USA) for 1 h. Nuclear counterstaining was performed with DAPI (S2110, Solarbio, Beijing, China). Fluorescent images were captured with a fluorescence microscope (Leica Dmi8 + DFC, Wetzlar, Germany).

### 5.19 Detection of Intracellular ROS, Lipid ROS, and Mitochondrial ROS

After appropriate treatment, cells were incubated with 10 µm DCFH‐DA (S0033, Beyotime Biotechnology, Shanghai, China), 10 µm C11 BODIPY (D3861, Invitrogen, Thermo Fisher Scientific, Waltham, MA, USA), and 1 µm MitoSOX Red (M36008, Invitrogen, Thermo Fisher Scientific, Waltham, MA, USA) for 30 min at 37°C in the dark. Nuclei were counterstained with DAPI. Fluorescence imaging was performed using a Leica Dmi8 + DFC fluorescence microscope (Wetzlar, Germany).

### 5.20 Measurement of MDA and GSH/GSSG Ratio

MDA content and GSH and GSSG levels were determined using an MDA assay kit (S0131, Beyotime Biotechnology, Shanghai, China) and GSH and GSSG assay kits (S0053, Beyotime Biotechnology, Shanghai, China), respectively. The optical densities at 532 and 412 nm were measured using a microplate reader (Spark, Tecan Group Ltd., Männedorf, Switzerland).

### 5.21 Mitochondrial Membrane Potential Analysis

A Mitochondrial Membrane Potential Detection Kit (C2006, Beyotime Biotechnology, Shanghai, China) was used according to the manufacturer's instructions. Images were obtained using a fluorescence microscope (Dmi8 + DFC; Leica, Wetzlar, Germany).

### 5.22 Statistical Analysis

Statistical analyses were conducted using GraphPad Prism version 9.0. Quantitative data are expressed as mean ± standard deviation. Comparisons between two groups were performed with an unpaired Student's *t*‐test, while differences among multiple groups were assessed using one‐way analysis of variance followed by Dunnett's multiple‐comparisons test. *P* < 0.05 was considered statistically significant. All experiments were independently repeated at least three times.

## Author Contributions

**Han‐jun Qin**: Conceptualization, Methodology, Writing – review&editing, Funding acquisition; **Si‐ying He**: Investigation, Formal analysis, Visualization, Data curation, Writing – original draft; **Ting‐hui Xiao**: Resources, Validation; **Da‐mao Dai**: Funding acquisition, Project administration; **Xin‐jia Hu**: Resources, Supervision; **Nan Jiang**: Conceptualization, Resources, Funding acquisition

## Funding

This work was supported by National Natural Science Foundation of China (No. 82502919), China Postdoctoral Science Foundation (No. 2024M752141), Jiangxi Provincial Natural Science Foundation (No. 20252BAC220051), Guangdong Provincial Basic and Applied Basic Research Fund Project (#2024A1515220069) and Shenzhen Science and Technology Research and Development Funds (#GJHZ20240218114506011).

## Conflicts of Interest

The authors declare no conflicts of interest.

## Supporting information




**Supporting File**: advs74115‐sup‐0001‐SuppMat.docx.

## Data Availability

The data that support the findings of this study are available on request from the corresponding author.
