# Supplementary material for: Lactylation Reprogramming in the Bone Infection Microenvironment Identifies PGK1 K361 as a Potential Therapeutic Target for Osteogenic Dysfunction
Source: Adv Sci (Weinh). 2026 Jan 28;13(19):e23583. doi: 10.1002/advs.202523583 (PMC13045457; doi:10.1002/advs.202523583)
Supplement: Supplementary file 1 — Supporting File: advs74115‐sup‐0001‐SuppMat.docx. [file ADVS-13-e23583-s001.docx]

Supporting Information

Lactylation Reprogramming in the Bone Infection Microenvironment Identifies PGK1 K361 as a Potential Therapeutic Target for Osteogenic Dysfunction

Han-jun Qin^1,2#^, Si-ying He^2#^, Ting-hui Xiao^1,2^, Da-mao Dai^3^*, Xin-jia Hu^1,2^*, Nan Jiang^4,5^*

^1^ Division of Orthopaedic Trauma, Department of Orthopaedic Surgery, Shenzhen People’s Hospital (The First Affiliated Hospital, Southern University of Science and Technology; The Second Clinical Medical College, Jinan University), Shenzhen, 518020, China.

^2^ Shenzhen Key Laboratory of Musculoskeletal Tissue Reconstruction and Function Restoration, Department of Orthopaedic Surgery, Shenzhen People's Hospital (The First Affiliated Hospital, Southern University of Science and Technology; The Second Clinical Medical College, Jinan University), Shenzhen, 518020, China.

^3^ Department of Plastic and Cosmetic Surgery, Shenzhen People's Hospital (The First Affiliated Hospital, Southern University of Science and Technology; The Second Clinical Medical College, Jinan University), Shenzhen, 518020, China.

^4^ Department of Trauma Emergency Center, Ganzhou Hospital-Nanfang Hospital, Southern Medical University, Ganzhou, 510515, China.

^5^ Division of Orthopaedics and Traumatology, Department of Orthopaedics, Nanfang Hospital, Southern Medical University, Guangzhou, 510515, China.

*Correspondence: Da-mao Dai [(handsomeddm@163.com)](mailto:(handsomeddm@163.com),)丨Xin-jia Hu ([hxjys888@hotmail.com](mailto:hxjys888@hotmail.com))丨

Nan Jiang ([hnxyjn@smu.edu.cn](mailto:hnxyjn@smu.edu.cn))

^#^Han-jun Qin and Si-ying He contributed equally to this work.

**
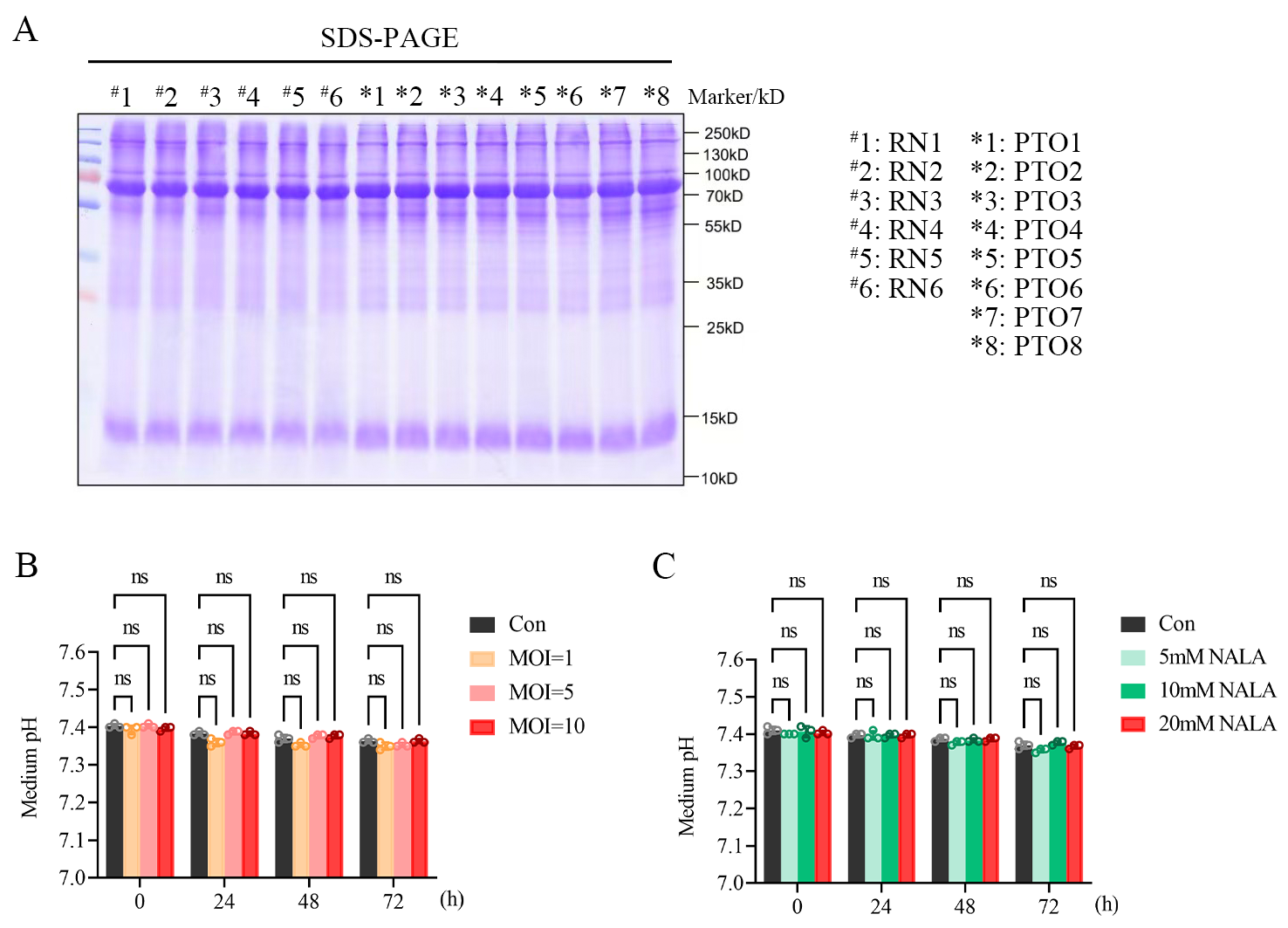
**

**Figure S1. Protein loading control and medium pH measurements.** (A) Coomassie Brilliant Blue-stained SDS-PAGE showing total protein loading uniformity across lanes; (B) culture medium pH across MOI conditions; and (C) culture medium pH under NALA treatments. Data represent mean ± SEM, *n* = 3 per group. Statistical significance was assessed using a one-way ANOVA with Dunnett's multiple-comparison test (B, C). ns: no significance.

**
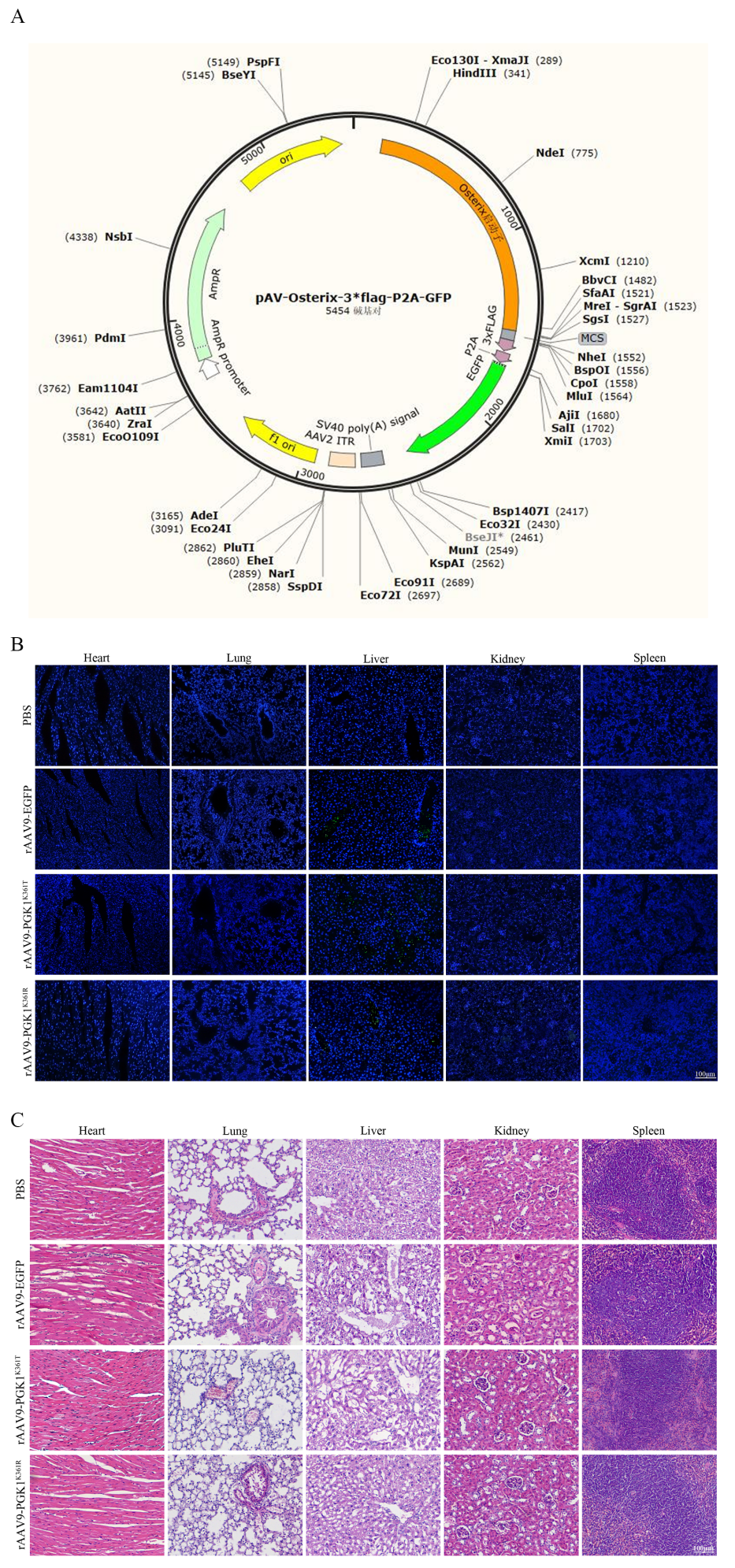
**

**Figure S2.** **rAAV9-Osx targeting validation and biosafety assessment.** (A) rAAV9-GFP under the osterix (*Osx*) promoter: bone-targeting validation; (B) IF staining of GFP in femur and liver. Scale bar, 100 μm; (C) H&E staining of major organs; Scale bar, 100 μm.

**
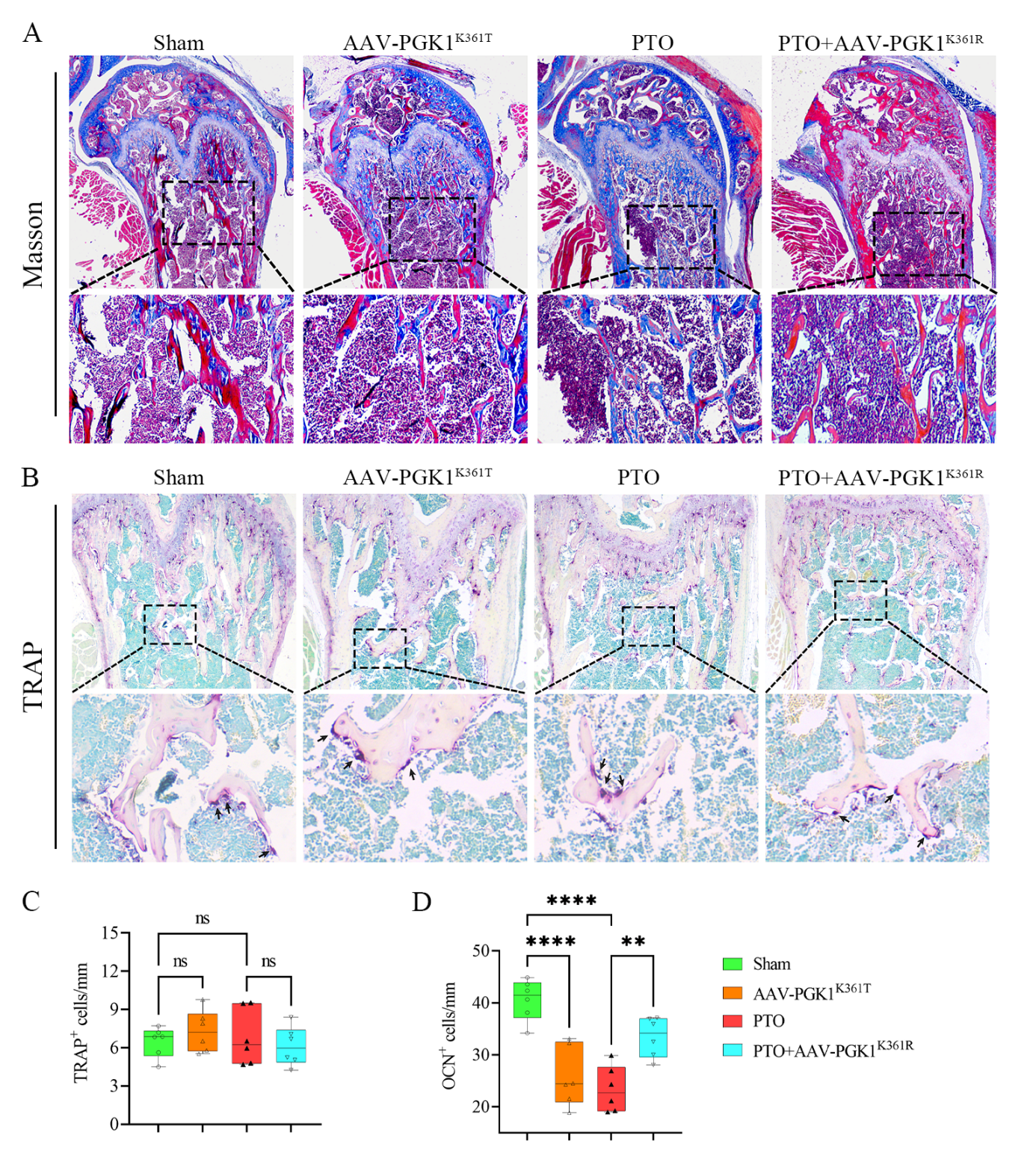
**

**Figure S3. Histological staining results and quantitative analyses.** (A) Representative images of Masson trichrome staining; Scale bar, 200 μm; magnified view, 100 μm; (B, C) Representative images and quantification of TRAP staining; Scale bar, 200 μm; magnified view, 100 μm; (D) Quantification of OCN staining from Figure. 4H. Data represent mean ± SEM, *n* = 6 per group. Statistical significance was assessed using a one-way ANOVA with Dunnett's multiple-comparison test (C, D). **P* < 0.05, ***P* < 0.01, ****P* < 0.001, *****P* < 0.0001, ns: no significance.

**
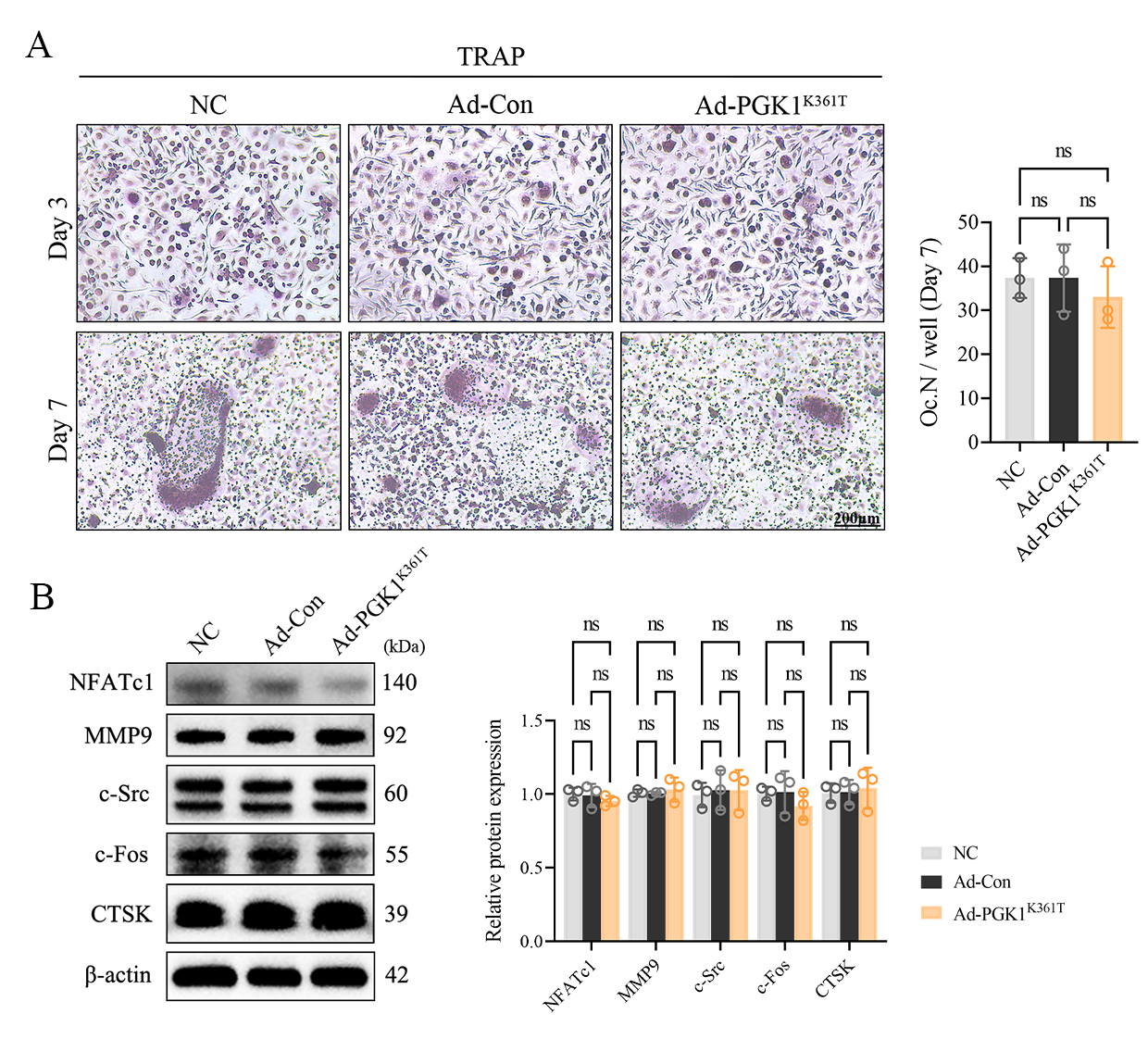
**

**Figure S4.** **Osteoclast differentiation assays and marker expression.** (A) Representative TRAP staining of BMDM-induced osteoclasts and quantitative analysis; (B) Western blotting and quantification of osteoclastogenesis-related proteins. Data represent mean ± SEM, *n* = 3 per group. Statistical significance was assessed using a one-way ANOVA with Dunnett's multiple-comparison test (A, B). ns: no significance.


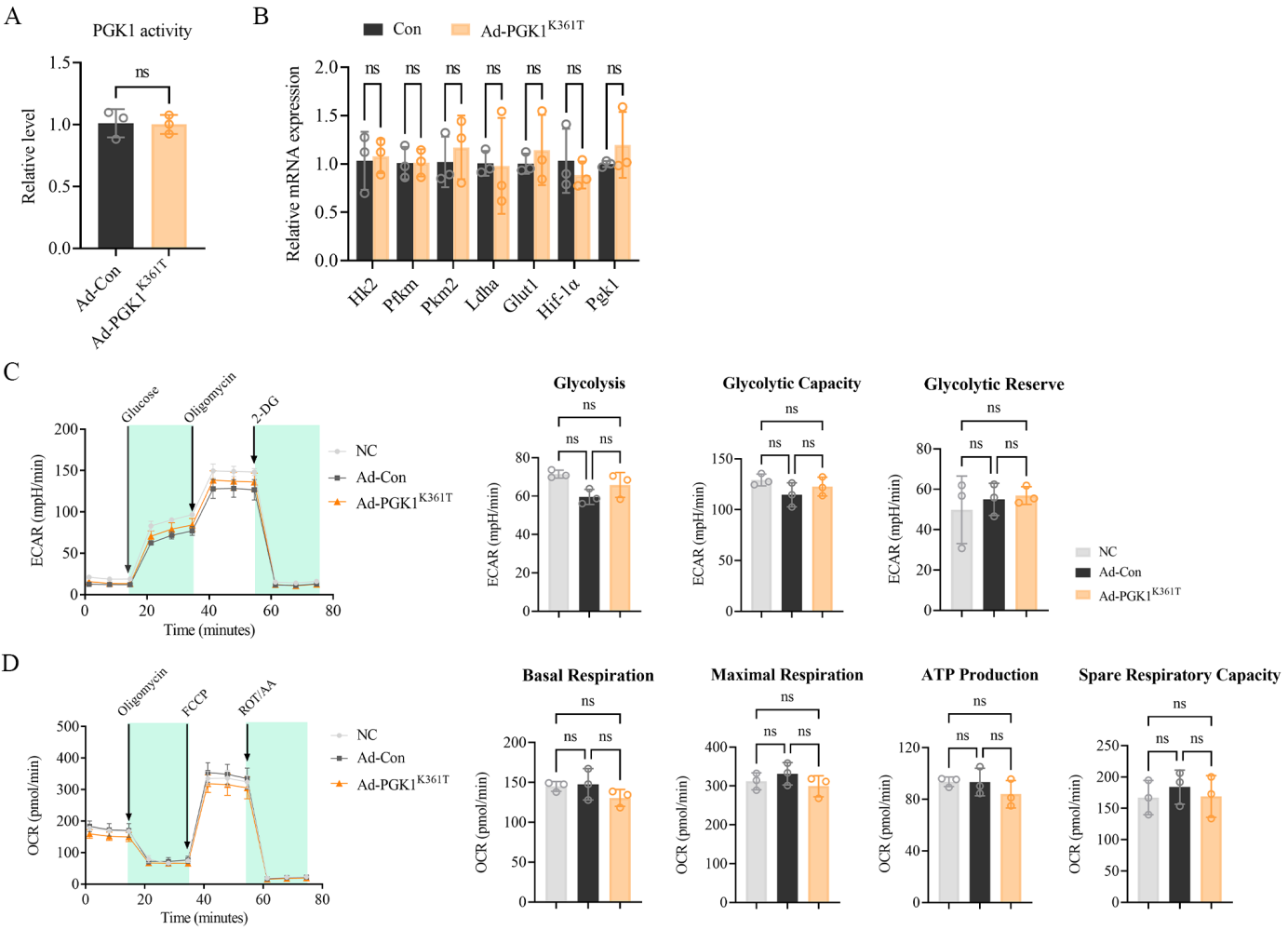

**Figure S5.** **Glycolysis-related readouts and bioenergetic profiling.** (A) PGK1 enzymatic activity after K361 lactylation; (B) Expression of glycolysis-related genes quantified using qPCR; (C) ECAR and OCR analysis. Statistical significance was assessed using unpaired Student's *t*-test (A, B) and one-way ANOVA with Dunnett's multiple comparison test (C, D). ns: no significance.

**
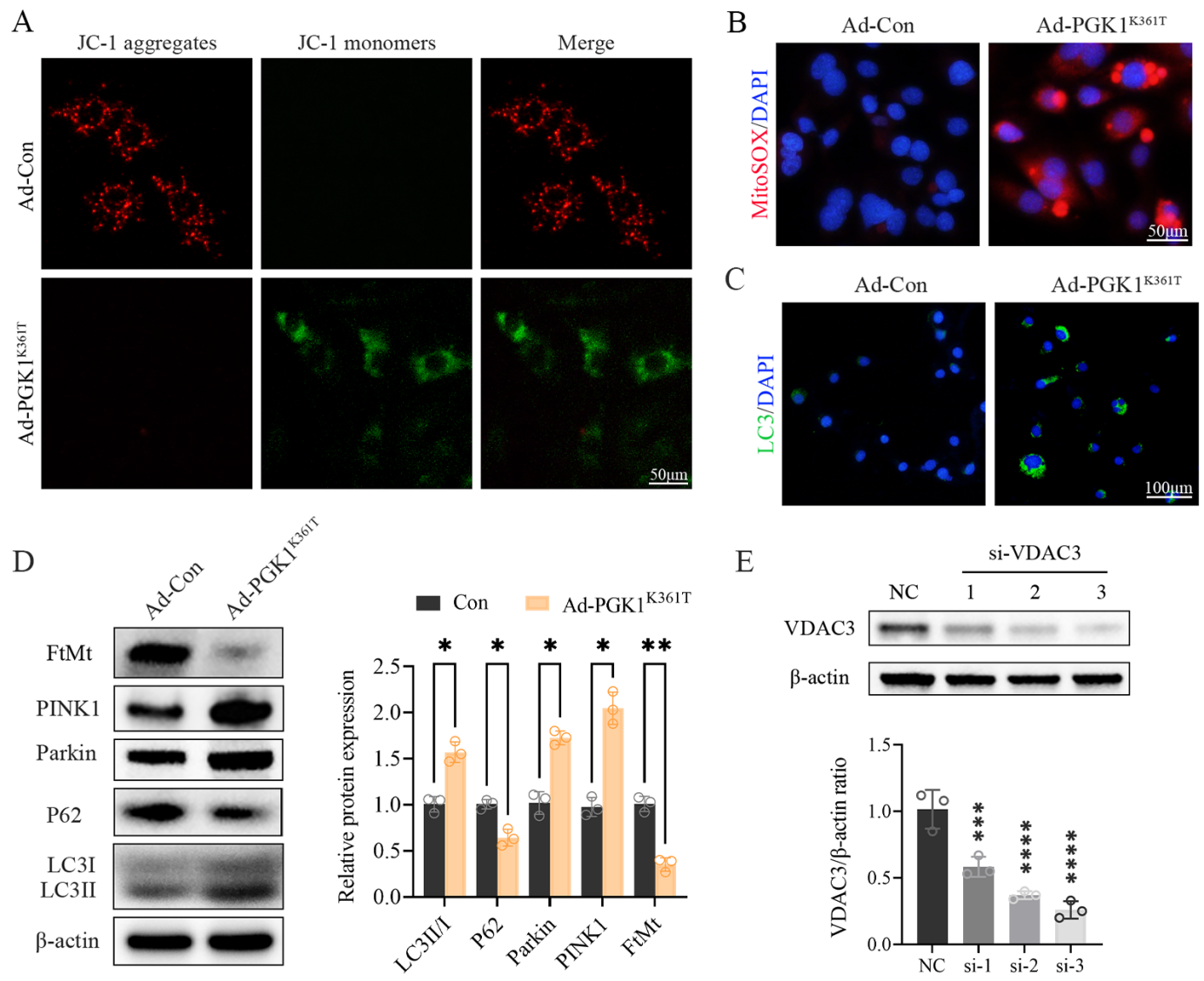
**

**Figure S6. Mitochondrial assays and VDAC3 knockdown efficiency.** (A–C) Representative images of mitochondrial membrane potential (JC-1), mitochondrial ROS (MitoSOX), and LC3 IF after Ad-PGK1K361T transfection; Scale bar, 50/100 μm; (D) Western blotting and quantification of mitophagy-related proteins after Ad-PGK1^K361T^ transfection. LC3II is quantified as the LC3II/LC3I ratio; P62, Parkin, PINK1, and FtMt are normalized to β-actin; (E) Western blotting and quantification of VDAC3 knockdown efficiency. Statistical significance was assessed using unpaired Student's *t*-test (D) and one-way ANOVA with Dunnett's multiple comparison test (E). **P* < 0.05, ***P* < 0.01, ****P* < 0.001, *****P* < 0.0001, ns: no significance.

**Supplementary Table 1. Antibodies and reagents**

| Products | Cat. No | Supplier |
| --- | --- | --- |
| Sodium L-lactate | 71718 | Sigma-Aldrich |
| Ferrostatin-1 | S7243 | Selleck |
| Mdivi-1 | S7162 | Selleck |
| Anti-L-Lactyl Lysine Rabbit mAb | PTM-1401RM | PTM |
| p300 Polyclonal antibody | 20695-1-AP | Proteintech |
| PGK1 Monoclonal antibody | 68035-1-Ig | Proteintech |
| Anti-xCT antibody [EPR8290(2)] | ab175186 | Abcam |
| GPX4 Monoclonal antibody | 67763-1-Ig | Proteintech |
| VDAC3 Polyclonal antibody | 55260-1-AP | Proteintech |
| Anti-Mitochondrial Ferritin antibody [EPR1797] | ab124889 | Abcam |
| PINK1 Polyclonal antibody | 23274-1-AP | Proteintech |
| PARK2/Parkin Polyclonal antibody | 14060-1-AP | Proteintech |
| P62/SQSTM1 Polyclonal antibody | 18420-1-AP | Proteintech |
| LC3 Polyclonal antibody | 14600-1-AP | Proteintech |
| MFN2 Polyclonal antibody | 12186-1-AP | Proteintech |
| MFN1 Polyclonal antibody | 13798-1-AP | Proteintech |
| DRP1 (C-terminal) Polyclonal antibody | 12957-1-AP | Proteintech |
| Beta Actin Monoclonal antibody | 60008-1-Ig | Proteintech |

**Supplementary Table 2. siRNA sequences**

| si-p300 | Forward: 5’-GGAAUUGGAGAUCAGCUUA-3’  Reverse: 5’-UAGCUGUAGUCCUAGAUGC-3’ |
| --- | --- |
| si-VDAC3 | Forward: 5’-GCUGCAUACUCACGUGAAUTT-3’  Reverse: 5’-AUUCACGUGAGUAUGCAGCTT-3’ |

**Supplementary Table 3. Primers for quantitative RT-PCR**

| Primers | Sequence (5’→3’) |
| --- | --- |
| Alp F | ATCTTTGGTCTGGCTCCCATG |
| Alp R | TTTCCCGTTCACCGTCCAC |
| Ocn F | GCTCTGTCTCTCTGACCTCA |
| Ocn R | TGGACATGAAGGCTTTGTCA |
| Hk2 F | CTACATGGAGGAGATGCGTAAT |
| Hk2 R | GCTTTGTGAAATCGATCAGGAT |
| Pfkm F | GACAGACTTTGAACACCGAATC |
| Pfkm R | CAATCTCGTACTTGGCTAGGAT |
| Pkm F | TATCATTGCCGTGACTCGAAAT |
